# Investigating the role of a conserved hydrophobic pocket of gp41 in the anti‐HIV activity of fusion inhibitors

**DOI:** 10.1002/pro.70593

**Published:** 2026-05-04

**Authors:** Daniel Polo‐Megías, Mario Cano‐Muñoz, Laura Sánchez‐Martínez, Sara Lestani, Christiane Moog, Thomas Decoville, María Carmen Salinas‐García, José Antonio Gavira, Ana Cámara‐Artigas, Francisco Conejero‐Lara

**Affiliations:** ^1^ Departamento de Química Física Instituto de Biotecnología y Unidad de Excelencia de Química Aplicada a Biomedicina y Medioambiente (UEQ), Facultad de Ciencias, Universidad de Granada Granada Spain; ^2^ Laboratoire d'ImmunoRhumatologie Moléculaire Institut National de la Santé et de la Recherche Médicale (INSERM) UMR_S 1109, Institut Thématique Interdisciplinaire (ITI) de Médecine de Précision de Strasbourg, Transplantex NG, Faculté de Médecine, Fédération Hospitalo‐Universitaire OMICARE, Fédération de Médecine Translationnelle de Strasbourg (FMTS), Université de Strasbourg Strasbourg France; ^3^ Vaccine Research Institute (VRI) Créteil France; ^4^ Laboratorio de Estudios Cristalográficos IACT‐CSIC Granada Spain; ^5^ Department of Chemistry and Physics University of Almería, Agrifood Campus of International Excellence (ceiA3), Research Center for Mediterranean Intensive Agrosystems and Agri‐Food Biotechnology (CIAIMBITAL) Almería Spain

**Keywords:** antivirals, calorimetry, coiled‐coils, fusion inhibitors, protein stability, small‐molecule inhibitors, X‐ray crystallography

## Abstract

Membrane fusion between HIV and host cells requires interaction between the N‐terminal and C‐terminal repeat regions (NHR and CHR) of the gp41 envelope subunit. A deep hydrophobic pocket (HP) on the surface of NHR is considered crucial in this interaction. Targeting the viral gp41 CHR with stabilized trimeric NHR peptides or chimeric proteins effectively inhibits HIV infection. However, the contribution of each specific structural element, particularly the HP, in this mode of inhibition remains unclear. Here, we describe three chimeric proteins that structurally mimic the full NHR region interacting intramolecularly with CHR segments of varying lengths, either covering or exposing the HP. The intramolecular NHR–CHR interaction strongly stabilized all the chimeras. Binding analysis using a CHR‐derived peptide and the hydrophobic probe 8‐anilino‐1‐naphthalene sulfonate (ANS) combined with X‐ray crystallography assessed the degree of exposure of the HP in the chimeras. Despite differences in HP accessibility, none of the chimeras displayed relevant inhibitory activity against several HIV‐1 strains, suggesting that an exposed HP alone is insufficient to disrupt the NHR–CHR interaction in a viral context. The crystal structure of the ANS–chimera complex revealed the binding pose of ANS within the HP, while the overall CHR–NHR interaction closely resembled the canonical post‐fusion six‐helix‐bundle structure. To our knowledge, this is the first crystallographic structure of a small molecule ligand independently bound to the HP. These findings provide insight into the role of the HP in NHR‐based fusion inhibitors and guide the design of new, more focused and effective HIV inhibitors.

## 1 INTRODUCTION

HIV infection continues to be a severe pandemic of enormous health and socioeconomic relevance, with more than 40 million people living with the virus and 1.3 million new infections in 2024 (UNAIDS Fact Sheet 2025). Despite massive efforts, an effective vaccine is still missing (Haynes et al. 2023), and current treatments rely on combinations of antiretroviral drugs that reduce viral load but do not eliminate the virus from a reservoir of infected cells (Saag et al. 2020). It is therefore necessary to continue the search for new strategies to develop effective antivirals.

HIV‐1 infects CD4+ immune cells by means of its envelope glycoprotein (Env), a trimer of heterodimers composed of gp120 and gp41 subunits (Chen 2019). Virus attachment begins with the binding of Env's gp120 to the cell's CD4 receptor. Subsequent binding between a secondary gp120 site and a coreceptor (CCR5 or CXCR4) triggers a series of conformational changes in the gp41 subunit that start with insertion of its fusion peptide (FP) in the cell membrane, followed by gp41 folding to form a post‐fusion structure, in which its N‐terminal and C‐terminal heptad repeat regions (NHR or HR1, and CHR or HR2, respectively) associate to form a highly stable six‐helix bundle (6HB) (Chan et al. 1997; Weissenhorn et al. 1997). This event brings together the cell and viral membranes, favoring their fusion, pore formation, and viral entry. However, the molecular details of the conformational transition of gp41 that drives membrane merging and fusion are still not fully understood. There is a broad consensus supporting the existence of a short lived pre‐fusion intermediate state, in which the CHR and NHR regions are transiently exposed. In this state, the NHR region allegedly forms an extended parallel coiled‐coil helical trimer, with three long hydrophobic grooves on the exposed surface of each pair of helices. To form the post‐fusion 6HB, the CHR regions must associate externally on top of the NHR grooves in an antiparallel orientation, acquiring helical structure. The pre‐fusion intermediate can be targeted by a variety of inhibitors, generically termed “fusion inhibitors,” including CHR‐derived peptides (Eron et al. 2004; Kilby et al. 1998), NHR‐based peptides and protein constructs (Bianchi et al. 2005; Crespillo et al. 2014; Louis et al. 2001; Root et al. 2001), antibodies (Miller et al. 2005; Sabin et al. 2010), nanobodies (Strokappe et al. 2019), and small‐molecule compounds (He et al. 2022; Qiu et al. 2019; Zhou et al. 2021), all of which block 6HB formation, membrane fusion and viral entry.

**FIGURE 1:**
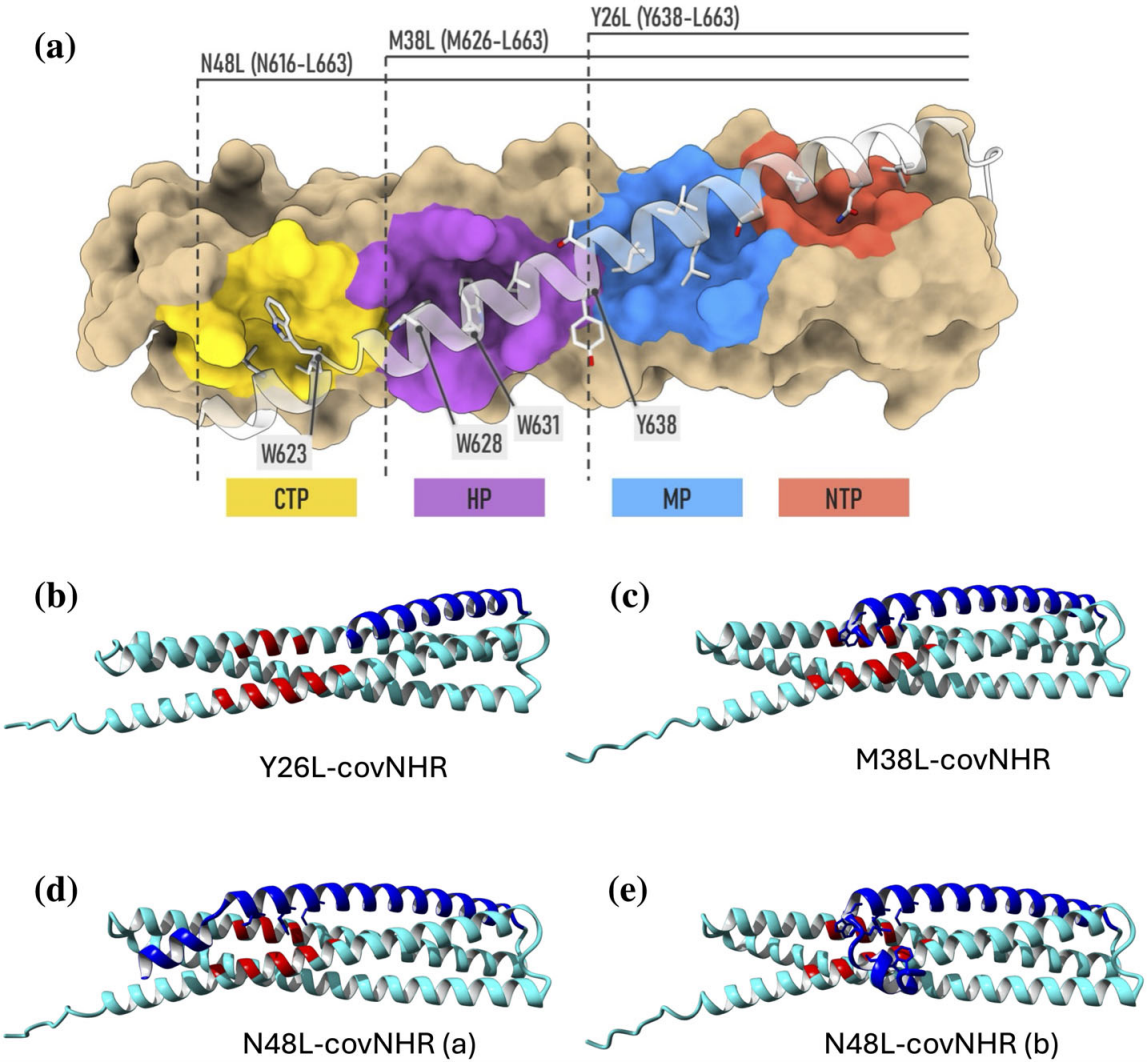
Design of the chimeric proteins. (a) Schematic model of the N48L‐covNHR chimera showing the covNHR surface, with the different NHR pockets labeled and highlighted in different colors, and the CHR region represented with semi‐transparent ribbon. The CHR segments of each chimera, along with their sequences in gp160 numbering are indicated by lines. The most relevant side chains interacting with the NHR pockets are represented with sticks. (b–e) Ribbon representation of the predicted structures of the three chimeras by AlphaFold3. (b) Y26L‐covNHR; (c) M38L‐covNHR; (d, e) N48L‐covNHR was predicted in two different conformations. The covNHR domain is colored in cyan with residues that form the HP highlighted in red. The CHR segments are colored in blue. The side chains of CHR residues interacting with the HP are represented with sticks.

The gp41 6HB post‐fusion structure has mainly inspired the design of different families of fusion inhibitors. On this basis, CHR‐derived peptides have strong antiviral activity due to their capacity to bind NHR and interfere with the endogenous NHR–CHR interaction and 6HB formation. Previous studies have shown that the strong NHR–CHR interaction energy results from multiple cooperative interactions involving several adjacent pockets along the NHR groove (Johnson et al. 2011; Jurado et al. 2020a). Among these, there is a highly conserved, prominent interaction on each NHR groove involving a deep hydrophobic pocket (HP), in which several hydrophobic side chains of CHR residues are inserted. This CHR motif, known as the “pocket‐binding motif” (PBM), is mainly composed of residues Trp628, Trp631, and Ile635 (Cano‐Muñoz et al. 2021; Chan et al. 1998). Due to its special features and high sequence preservation among HIV‐1 strains, the HP has become a highly pursued target for the design of inhibitors (Rad et al. 2018). However, other pockets upstream of the HP in the NHR groove are also important in 6HB stability. These include a shallow hydrophobic pocket that was called “middle pocket” (MP) and a preserved N‐terminal polar pocket (NTP) (Figure 1a) (Jurado et al. 2020a).

Among CHR peptides, T20 (enfuvirtide®), corresponding to Env gp41 residues 638–673, is the first and only FDA‐approved fusion inhibitor so far (Kilby et al. 1998). Unfortunately, enfuvirtide treatment rapidly develops resistant viral strains with mutations mostly concentrated in the 547–556 NHR segment (Greenberg 2004; Reeves et al. 2005) that impair T20 binding to NHR. In addition, T20 has a limited inhibitory potency because it does not contain the PBM; it only encompasses the C‐terminal half of CHR and a tryptophan‐rich motif corresponding to the membrane‐proximal external region (MPER) of gp41. It is accepted that T20 binds to the MP and NTP pockets of the N‐terminal half of NHR and to the membrane via its hydrophobic MPER region, although it has been suggested that T20 may have other binding targets (Zhang et al. 2019). CHR peptides containing the PBM exhibit higher inhibitory potency and a greater genetic barrier to resistance than T20 (Chan et al. 1998; Eron et al. 2004; Feo and Weiss 2012; He et al. 2008). Moreover, antibodies binding to the HP have shown potent neutralizing activity (Miller et al. 2005; Sabin et al. 2010; Sun et al. 2024), but it was found that the neutralizing activity of these mAbs is enhanced in their ScFv or Fab form compared to their full IgG, suggesting steric impairment to access the HP (Eckert et al. 2008; Sabin et al. 2010).

Mirroring the above approach, NHR‐derived peptides can also inhibit HIV‐1 fusion by targeting CHR. However, as isolated peptides, they have a much lower antiviral activity due to their higher hydrophobicity, lower solubility, and propensity to aggregate (Eckert and Kim 2001). However, it was shown that their inhibitory activity can be strongly improved by stabilizing a trimeric NHR in coiled‐coil conformation (Dwyer et al. 2008; Wild et al. 1994). Accordingly, a variety of engineered NHR constructs with a stable trimeric conformation that exposes at least part of the NHR groove have been developed as potent fusion inhibitors (Bianchi et al. 2005; Chen et al. 2010a; Chu et al. 2015; Crespillo et al. 2014; Louis et al. 2001; Root et al. 2001; Walsh et al. 2015). This family of inhibitors shows a higher genetic barrier to the development of resistance because NHR is more sequence‐conserved than CHR, although prolonged treatment with NHR constructs selects for resistant viruses with mutations at both NHR and CHR that enhance 6HB stability (Feo and Weiss 2012).

It has been generally accepted that, to be an effective inhibitor, an NHR‐based construct should contain the HP to bind to the PBM of CHR in viral gp41. However, during the Env‐mediated fusion process, gp41 NHR and CHR may not be equally accessible. Moreover, different CHR motifs could be differently exposed during the gp41 conformational change. In the native Env structure, the PBM is engaged in a tryptophan clamp that grabs the gp120 subunit and stabilizes the prefusion conformation (Lee et al. 2016). To unfasten this clamp and dissociate gp120, Env needs to be activated by receptor and coreceptor binding (Pancera et al. 2014). These events release the PBM, which subsequently binds to the endogenous HP upon formation of the 6HB, leaving a narrow time window for NHR‐based inhibitors to recognize and capture the PBM, thereby kinetically limiting their inhibitory activity (Kahle et al. 2009; Steger and Root 2006).

Early designs fused NHR segments of different lengths to trimerization motifs or “foldons” to stabilize their trimeric coiled‐coil structure (Chen et al. 2010a; Eckert and Kim 2001), or were further stabilized by intermolecular crosslinks (Bianchi et al. 2005; Louis et al. 2003). All these constructs contained the NHR segment that forms the HP, which was described as the minimal NHR motif showing inhibitory activity (Bianchi et al. 2005; Eckert and Kim 2001). To avoid the need for trimerization and chemical crosslinking, single‐chain constructs were designed, including 5‐helix (Root et al. 2001) or covNHR (Crespillo et al. 2014), each displaying a single, complete NHR groove. Deriving from the latter, truncated covNHR variants were produced exposing each half of the NHR groove (Jurado et al. 2020b). CovNHR‐N constructs, displaying the N‐terminal NHR half, showed mid‐to‐low nM HIV‐1 inhibitory activity, even though they do not contain the HP (Cano‐Muñoz et al. 2022; Jurado et al. 2020b; Polo‐Megías et al. 2025). Importantly, a strong correlation between structural stability of the trimeric state and inhibitory potency was consistently found (Cano‐Muñoz et al. 2022; Dwyer et al. 2008; Eckert and Kim 2001).

Despite extensive prior research, the precise role of the HP in NHR‐based fusion inhibitors is still not fully understood. To give insight into this question, we designed three chimeric proteins by fusing CHR segments of different lengths to the N‐terminus of the full‐length covNHR protein (Jurado et al. 2019). The length of the CHR segment was adjusted to cover two, three, or four pockets of the NHR groove. The chimeras were produced recombinantly and structurally characterized in comparison with the covNHR protein, which exposes the whole NHR groove. The in vitro inhibitory activity of two chimeras, one exposing the HP and another with all NHR pockets covered, was compared with that of the covNHR proteins. Additionally, the crystal structures of a chimera exposing the HP in its free form and in complex with the hydrophobic probe 8‐anilino‐1‐naphthalene sulfonate (ANS) were determined. To our knowledge, we report here the first high‐resolution crystallographic structure of a small‐molecule compound independently bound to the HP. The results presented here give insight into the role of the HP in NHR‐mimics, as anti‐HIV‐1 fusion inhibitors.

## 2 RESULTS

### 2.1 Design of three chimeric proteins based on covNHR

Three chimeric proteins were modeled using the high‐resolution structure of covNHR in complex with the CHR‐derived peptide C34 (gp160 residues W628‐L661) (PDB code 6R2G) (Jurado et al. 2019) as a template. We first modeled a chimeric protein by fusing the CHR peptide C‐terminus to the N‐terminus of the covNHR protein using a four‐residue loop. Then, we modified the length of the CHR segment to cover two, three, or four NHR pockets (Figure 1a). This CHR–NHR arrangement resembles that used in previous studies, with the main difference being that our chimeric constructs were designed as single polypeptide chains, whereas previous ones were composed of three identical chains (Chu et al. 2015; Stewart et al. 2007; Stewart et al. 2010). One of the chimeras (named Y26L‐covNHR) included the CHR segment Y638‐L663 to cover only the NTP and the MP, while leaving exposed the HP and a minor C‐terminal pocket (CTP); a second chimera (named M38L‐covNHR) contained an N‐terminally extended CHR sequence M626‐L663 that covers the NTP, MP, and HP pockets; and finally, a third chimera (named N48L‐covNHR) included an elongated CHR peptide N616‐L663 that allegedly would cover completely the NHR groove. In this later chimeric protein, we initially assumed that the N‐terminal segment N616‐N625 would also cover the CTP, although no experimental structure is available for this interaction. The amino acid sequences are listed in Table S1, Supporting Information.

The structures of the chimeras were predicted using the AlphaFold3 server (https://alphafoldserver.com/; Abramson et al. 2024) (Figure 1b–e). The predicted structures of Y26L‐covNHR and M38L‐covNHR showed a high similarity with the crystallographic structure of the complex between covNHR and C34 (Jurado et al. 2019), reproducing very accurately the CHR–NHR interactions observed in the post‐fusion 6HB structure. However, in the case of N48L‐covNHR, only a fraction of the predicted models showed interaction of the N‐terminal part of CHR with the CTP, whereas in the other models, the Asn2‐Met12 segment (N616‐M626 in Env gp160 sequence) departs from the NHR groove and adopts a hook‐like structure, clustering the Trp9 (W623) and Met12 (M626) sidechains against NHR hydrophobic residues near the HP. In the M38L‐covNHR chimera, the N‐terminal Met‐Thr residues also adopt a hook‐like structure. In this case, the Met1 (M626) side chain stacks against Trp184 indole group (Trp571 in gp160). A similar hook structure that helps to stabilize the 6HB has been previously described for the CP621‐652 peptide in complex with the NHR peptide T21 (Chong et al. 2012).

### 2.2 Production and biophysical characterization of the chimeras

The three chimeras were cloned into commercial expression vectors, including a C‐terminal His‐tag to facilitate purification. The proteins were overexpressed in *E. coli* and purified using Nickel‐Tag Affinity chromatography and cation‐exchange chromatography, as described in section 4.1. The three proteins were highly soluble and behaved as monomers in solution, as indicated by dynamic light scattering (DLS) measurements (Figure S1 and Table S2), with no evidence of formation of oligomers or large aggregates. Only the Y26L‐covNHR chimera at pH 7.4 showed a slightly higher hydrodynamic radius than the other two, suggesting either a weak self‐association propensity or a more expanded structure.

**FIGURE 2:**
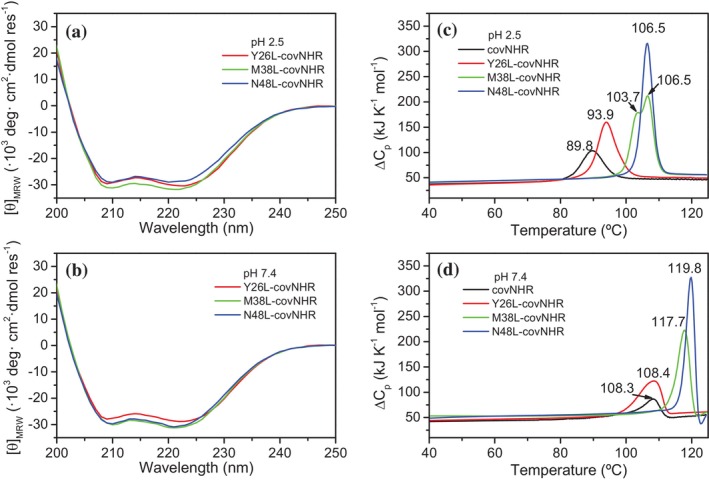
Structure and stability of the chimeras. (a, b) Far‐UV CD spectra of the chimeras measured at pH 2.5 in 50 mM glycine buffer (a) and at pH 7.4 in 50 mM sodium phosphate buffer (b). Data are normalized as mean‐residue molar ellipticity. (c, d) DSC thermograms showing the thermal unfolding of the chimeras at pH 2.5 (c) and pH 7.4 (d). Buffers are the same as in (a, b). The temperatures of the maximum of each peak are indicated next to each curve.

The circular dichroism (CD) spectra in the far‐UV wavelength range (200–260 nm) indicated α‐helical secondary structures for the three chimeras at both acidic and physiological pH (Figure 2a,b). The number of residues in α‐helix, estimated from the mean‐residue ellipticity at 222 nm (Luo and Baldwin 1997), was higher than that of the covNHR protein, indicating that the CHR segments acquire helical conformation in the chimeras, whereas, as isolated peptides they are mainly unstructured (Jurado et al. 2020a; Jurado et al. 2020b). In addition, the extra α‐helical content correlated well with the length of the CHR segment present in each chimera (Table S2).

The thermal unfolding profiles were measured by differential scanning calorimetry (DSC) at acidic and physiological pH (Figure 2c,d). The unfolding of the three chimeras was partially reversible at pH 2.5, but fully irreversible at pH 7.4 on a second consecutive scan. The increments in the temperature of unfolding (*T*_m_) and in the unfolding enthalpy (Δ*H*_m_) indicated a strong stabilization produced by the presence of the CHR segments compared to covNHR, and correlated well with the CHR segment length (Table S2). These results clearly indicate that the CHR regions fold into a helical conformation and tightly interact with the NHR groove in all the chimeras.

### 2.3 Analysis of binding of HP ligands

**FIGURE 3:**
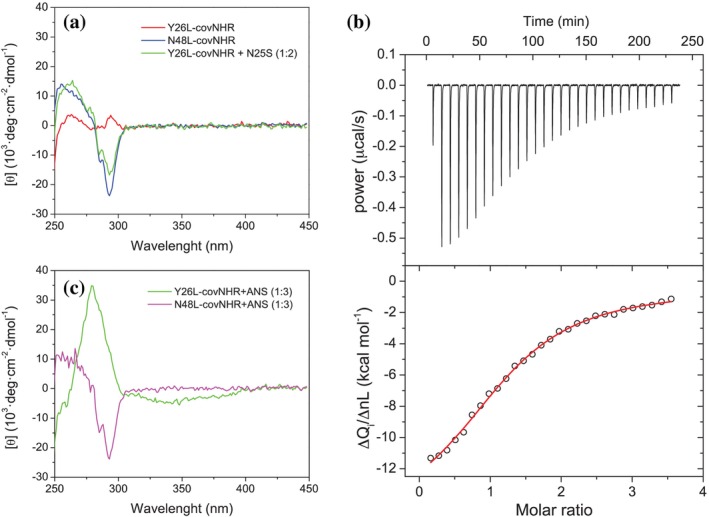
HP exposure in the chimeras. (a) Near‐UV CD spectra of Y26L‐covNHR, N48L‐covNHR, and Y26L‐covNHR in the presence of a 2:1 molar excess of N25S peptide. Ellipticity data were normalized per mole of protein. (b) ITC titration of Y26L‐covNHR with N25S peptide. The upper panel shows the experimental thermogram corrected from the baseline. In the lower panel, the symbols represent the normalized heats per mol of added peptide as a function of the peptide to protein molar ratio. The solid line corresponds to the best fit using a model of independent and equivalent binding sites. (c) Near‐UV CD spectra of Y26L‐covNHR and N48L‐covNHR in the presence of a 3:1 excess of ANS.

To ascertain the degree of exposure of the HP, we recorded near‐UV CD spectra (250–450 nm) of the Y26L‐covNHR and N48L‐covNHR chimeras (Figure 3a). The N48L‐covNHR chimera showed a prominent negative ellipticity band at 292 nm, which is characteristic of the insertion of two CHR tryptophan side chains onto the HP (Env residues Trp628 and Trp631) forming a cluster with the side chain of Trp571 (Crespillo et al. 2014). This indicated that the PBM in the CHR segment interacts with the HP in this chimera. In contrast, the Y26L‐covNHR chimera showed an insignificant CD signal intensity in this wavelength range, indicating that its aromatic side chains are fully solvent‐exposed. The only tryptophan residue in this chimera is Trp173 at the HP (W571 in gp160 sequence numbering). Thus, the absence of CD signal intensity suggested an exposed HP in the Y26L‐covNHR chimera, as expected.

Then, we recorded the CD spectrum of Y26L‐covNHR mixed with a 2:1 excess of the CHR peptide N25S (CHR residues N616‐S640), which contains the PBM. The spectrum of the mixture showed the same sharp negative band at 292 nm as observed for N48L‐covNHR, indicating that the peptide could access and bind the HP (Figure 3a). We measured the binding affinity between Y26L‐covNHR and the N25S peptide by isothermal titration calorimetry (ITC) (Figure 3b). The resulting dissociation constant, *K*_d_ = 3.3 ± 0.8 μM (Table S3), was very similar to that measured previously for the binding of the same peptide to the covNHR protein (Jurado et al. 2020a), and to the truncated covNHR‐C miniprotein that contains only the HP and the CTP (Jurado et al. 2020b). This confirmed that the HP of the Y26L‐covNHR chimera is fully exposed and capable of interacting with the PBM in a CHR peptide.

We previously reported that the fluorescent probe 8‐anilino‐naphthalene‐1‐sulfonic acid (ANS) can interact specifically with the HP (Cano‐Muñoz et al. 2021). The HP showed a dual ANS binding mode, depending on whether the remainder of the NHR groove was free or occupied by a CHR peptide. Thus, we recorded near‐UV CD spectra of Y26L‐covNHR and N48L‐covNHR in the presence of ANS at a 3:1 molar ratio (Figure 3c). In the presence of excess ANS, the CD spectrum of N48L‐covNHR remained invariant, indicating that ANS cannot interact with the chimera and does not compete with the NHR–CHR interaction at the HP. In contrast, the CD spectrum of Y26L‐covNHR in the presence of ANS showed a sharp and positive band at 280 nm and a broad negative band centered at about 342 nm. These bands had an identical shape but higher intensity than those previously described for ANS bound to the HP of covNHR when a CHR peptide Y24L (Env gp160 residues Y638‐L661) was occupying the NTP and MP pockets (Cano‐Muñoz et al. 2021). These results further confirmed that the HP of Y26L‐covNHR is exposed and available for ANS binding, whereas PBM of the intramolecular CHR segment completely occludes the HP of N48L‐covNHR.

### 2.4 HIV‐inhibition assays

**FIGURE 4:**
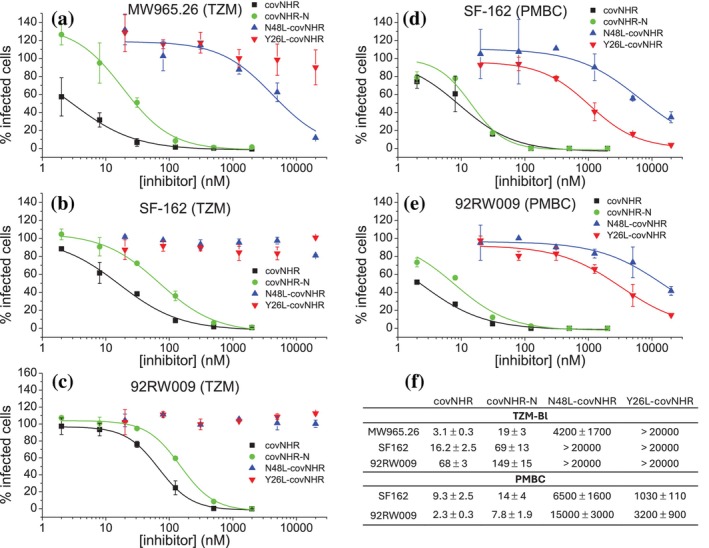
Inhibitory activity against cell infection by HIV‐1. TZM‐Bl (a–c) or PMBC (d, e) cells were infected with HIV‐1 pseudoviruses (a, b) or primary isolates (c–e) in presence of covNHR proteins or CHR‐covNHR chimeras. The percentage of infected cells relative to the controls are plotted against the inhibitor concentration. Data correspond to mean values ± SD from duplicates. The lines represent the best fits using a Hill's sigmoidal function. (f) IC_50_ values derived from the fits in nanomolar units.

To investigate the role of the exposed HP on the HIV‐1 inhibitory activity of NHR‐based chimeras, we compared the capacity of Y26L‐covNHR and N48L‐covNHR to inhibit HIV‐1 infection in vitro using the conventional single‐cycle TZM‐Bl assay with two pseudoviruses (SF‐162 of clade B and MW965.26 of clade C) and a Tier 2 primary isolate 92RW009 (clade A). The inhibitory activity of the chimeras was compared with the covNHR protein (fully exposing the whole NHR groove, including the HP) and with a highly stable covNHR‐N miniprotein (devoid of the HP and exposing only the N‐terminal half of NHR, that is, the NTP and MP pockets). This miniprotein was strongly stabilized by two disulfide bonds and three core mutations (Polo‐Megías et al. 2025). As shown in Figure 4, in single‐cycle assays, N48L‐covNHR showed only very weak activity against the easy‐to‐neutralize MW965.26 pseudovirus and no detectable activity against the other viruses. At the same time, the Y26L‐covNHR chimera exhibited no measurable activity against any of the three viruses, despite exposing the HP and being able to bind the PBM contained in the N25S peptide. Conversely, both covNHR and covNHR‐N proteins showed potent inhibitory activity, with the complete covNHR protein showing the highest inhibitory potency in these assays.

We also performed PBMC‐based inhibition assays with the SF‐162 and 92RW009 primary isolates (virus stocks produced in PBMCs). In this case, we observed weak inhibitory activities for the two chimeras, slightly higher for Y26L‐covNHR than for N48L‐covNHR, both with IC_50_ values in the μM range. In contrast, the covNHR and covNHR‐N proteins showed similarly potent nM activities against the two viruses, without any toxicity, even though the latter miniprotein does not contain the HP.

These results demonstrated that an exposed HP alone is insufficient to confer fusion inhibitory activity in these NHR‐based chimeras. Moreover, we also showed that potent inhibitory potency can be achieved with an NHR‐based inhibitor devoid of the HP. The weak activities of the chimeras may be attributable to an exchange between intramolecular binding of the NHR groove of the chimera with its own CHR segment and intermolecular binding with the viral CHR, perhaps favored by the membrane environment, as previously suggested (He et al. 2024). This exchange would be more favored in Y26L‐covNHR, which contains a shorter CHR segment and therefore weaker internal CHR–NHR interactions.

### 2.5 X‐ray crystallography

**FIGURE 5:**
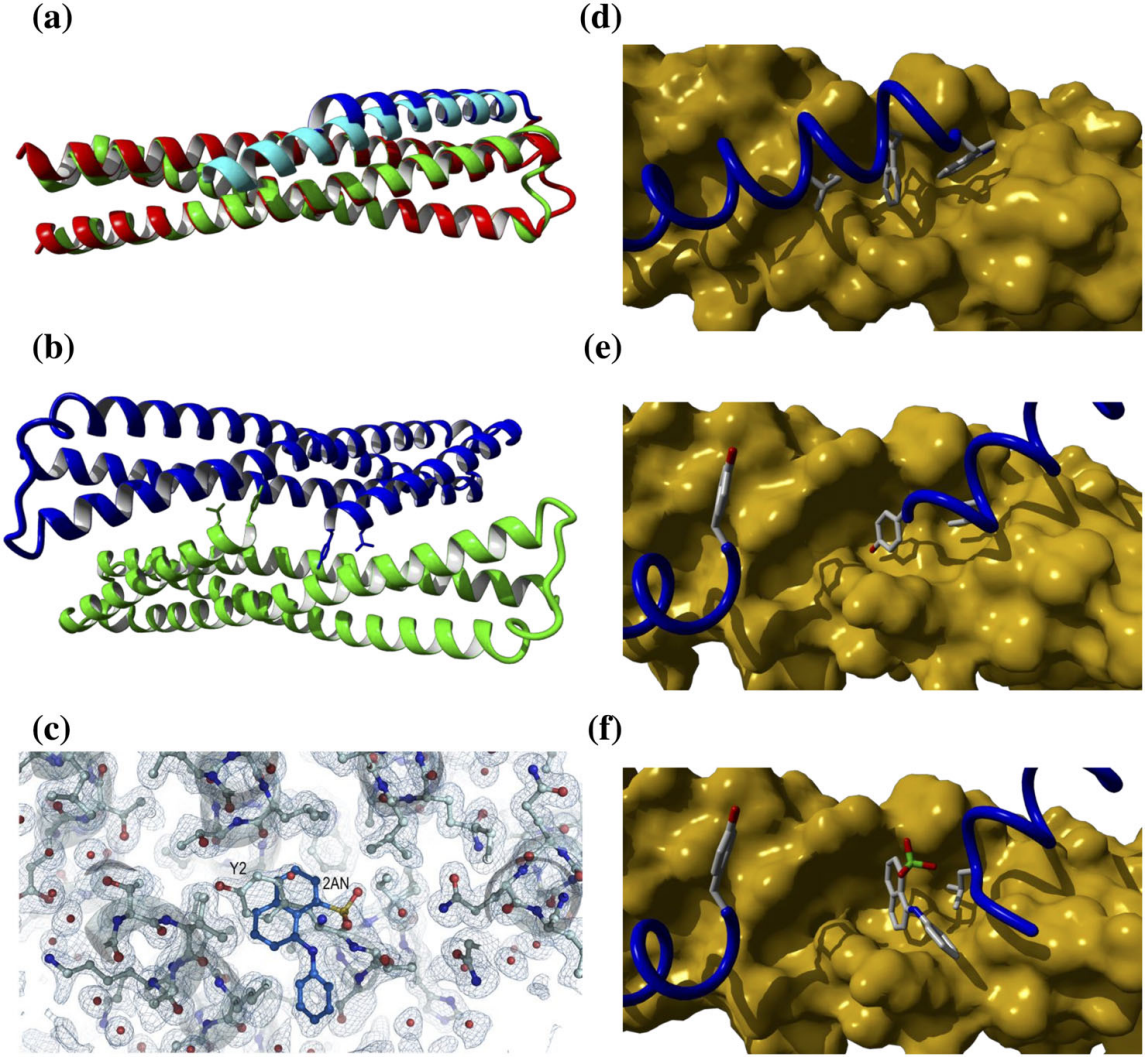
Crystal structure of Y26L‐covNHR in the absence and in the presence of ANS. (a) Structural alignment of Y26L‐covNHR in the C2_1_ crystal with the covNHR:C34 complex (PDB entry 6R2G); (Jurado el al. 2019). (b) Arrangement of Y26L‐covNHR monomers in the C2_1_ crystal polymorph. Tyr2 and Leu5 side chains inserted into the HP of the opposite monomer are represented with sticks. (c) 2Fo‐Fc electron density map, contoured to 1*σ* showing the region of the HP of Y26L‐covNHR in the P_1_ crystal polymorph. ANS molecule was represented in sticks (colored in blue) and the protein was represented in cartoon and sticks (pale cyan). The electron density was identified using a polder map to correspond to an ANS molecule modeled in half of the molecules and the amino terminal Tyr2 in the other half of the molecules of Y26L‐covNHR. Both were modeled bound to the HP with partial occupancy. Water molecules are colored in red. (d–f) Molecular surfaces of the covNHR moiety showing the HP in three different structures: (d) The covNHR:C34 complex (PDB entry 6R2G) showing the interaction of the PBM. Sidechains of Trp628, Trp631, and Ile635 are shown with sticks. (e) The Y26L‐covNHR chimera in the C2_1_ crystal polymorph (PDB entry 9SJQ), showing the Tyr2 and Leu5 side chains with sticks. (f) the Y26L‐covNHR chimera with bound ANS in the P_1_ crystal polymorph (PDB entry 9SJP), showing the ANS molecule and Leu5 side chain with sticks.

Although the crystallization conditions of the three chimeras were thoroughly screened using commercial kits, only well‐diffracting crystals of the Y26L‐covNHR chimera were obtained (Table S4), allowing its structure determination (PDB entry 9FED). A comparison of the experimental crystal structure coordinates with those of the computationally designed model revealed only minor differences. Structural alignment between Y26L‐covNHR and the covNHR:C34 complex (PDB entry 6R2G; Jurado et al. 2019) resulted in a global RMSD for the equivalent C_α_ atoms of 1.00 Å (Figure 5a). The highest RMSD values (>3 Å) were observed at the loops and the N‐ and C‐termini, as expected (Figure S2). The N‐terminal Met is not visible in the electron density map, and the following seven residues of the CHR segment, Tyr2‐Ser8 (Y638‐S644 in Env gp160 sequence numbering), are slightly lifted from the NHR groove compared to the covNHR:C34 complex. The remainder of the CHR region establishes intramolecular contacts with the NHR groove that are almost identical to those seen in the covNHR:C34 complex. The HP does not show any intramolecular contact in Y26L‐covNHR, and its structure and dimensions are similar to those of the covNHR:C34 complex, except for subtle rearrangements of some side chains. However, the HP of each Y26L‐covNHR monomer is involved in intermolecular contacts in the crystal, as described in more detail below (Figure 5b).

To investigate the binding mode of ANS to the HP, we co‐crystallized Y26L‐covNHR in the presence of an 8:1 excess of ANS, close to saturation, as determined by ITC measurements (see section 2.7). Two different high‐quality crystal polymorphs were obtained under the same crystallization conditions (0.2M ammonium sulfate, 30% w/v PEG 8000, 0.1M MES, pH 6.5). One polymorph belongs to the C2_1_ space group and contains only one molecule of the chimera in the asymmetric unit (Table S4; PDB entry 9SJQ). The electron density maps did not show any electron density corresponding to ANS molecules bound to the protein. Although the crystallization conditions and the cell parameters of these crystals are different from those of the crystals obtained in the absence of ANS, the overall structure of the chimera is essentially the same. However, the crystals obtained in the presence of ANS diffracted to a higher resolution (1.5 Å) than those obtained in its absence (2.3 Å). In both crystal structures, symmetry‐related molecules establish intermolecular contacts mediated by Tyr2 (Y638) and Leu5 (L641) side chains of the CHR segment from one molecule, and the HP from the opposite (Figure 5b). Remarkably, these two sidechains are inserted in the HP in very similar positions to the two CHR tryptophan sidechains in the covNHR:C34 complex (Figure 5d,e; see Supplementary Appendix S1, Figure S3, and Table S5 for additional details of the interactions between the monomers in the crystal).

The second crystal polymorph obtained in the presence of ANS belongs to P_1_ space group and diffracted at 1.15 Å resolution (Table S4; PDB entry 9SJP). Two molecules of the chimera were modeled in the asymmetric unit, forming a dimer. In this dimer, each monomer is associated with the other in an arrangement almost identical to that observed in crystals of the C2_1_ space group (Figure S4a), although with slightly different conformations (Figure S4b). However, in the HP, the electron density was considerably larger than that corresponding to a bound tyrosine side chain, suggesting the presence of bound ANS. To confirm that the ANS ligand was specifically bound to the HP in the crystal, we generated a polder map using Phenix (Liebschner et al. 2017). A polder map is an omit map that excludes the bulk solvent in the vicinity of the omitted region. This way, weak electron densities, which can be obscured by bulk solvent modeling, become more clearly visible. The density in this map, besides the presence of an ANS molecule at this position, also shows density corresponding to the Tyr2 side chain (Figure S5).

The polder maps showed that the electron density agrees with the placement of an ANS molecule bound to HP, as well as with the presence of Tyr2 (Y638) from the second molecule modeled in the asymmetric unit (Figure 5c). The electron density maps represent the average of symmetry‐related protein molecules in the crystal. In the context of the whole crystal, some protein molecules have only the ANS molecule bound to the HP, while others have only Tyr2 bound. To determine the fraction of molecules in the crystal with ANS or Tyr2 bound to the HP, we have refined the occupancy of the ANS molecule. For this purpose, we have performed several alternating cycles of B‐factor and occupancy refinement with phenix.refine. Several combined cycles were performed until the B‐factor and occupancy values did not change. Accordingly, in this crystal polymorph, ANS is bound to the HP in about half of Y26L‐covNHR molecules, whereas in the remainder a similar position in the HP is occupied by the Tyr2 (Y638) side chain of the opposite monomer in a very similar conformation as in the crystal polymorph of C2_1_ space group (Figure 5e). When ANS is bound, the first three CHR residues of the opposite monomer become displaced from the HP to allow for ANS entry. The naphthalene group of ANS fits in the HP in an orientation perfectly matching that of the indole group of CHR Trp631 in the covNHR:C34 complex (Figure 5f), establishing hydrophobic interactions with the pocket and laterally stacking with the Leu5 (L641) side chain of CHR segment of the opposite monomer. The sulfonate group of ANS forms hydrogen bonds with Leu5 (L641) and Ile6 (I642) amide groups of the CHR backbone, while the phenyl ring of the aniline moiety stacks laterally between the side chains of Lys65 (K574) and Gln68 (Q577) of the HP (see Supplementary Appendix S1, Figure S6, and Table S6). All these interactions highlight a strong binding potential of the HP, which also plays an important role in crystal contacts. To our knowledge, this structure is the first reported crystallographic structure of a small‐molecule compound bound independently to the HP.

### 2.6 Analysis of dimerization of the chimera in solution

**FIGURE 6:**
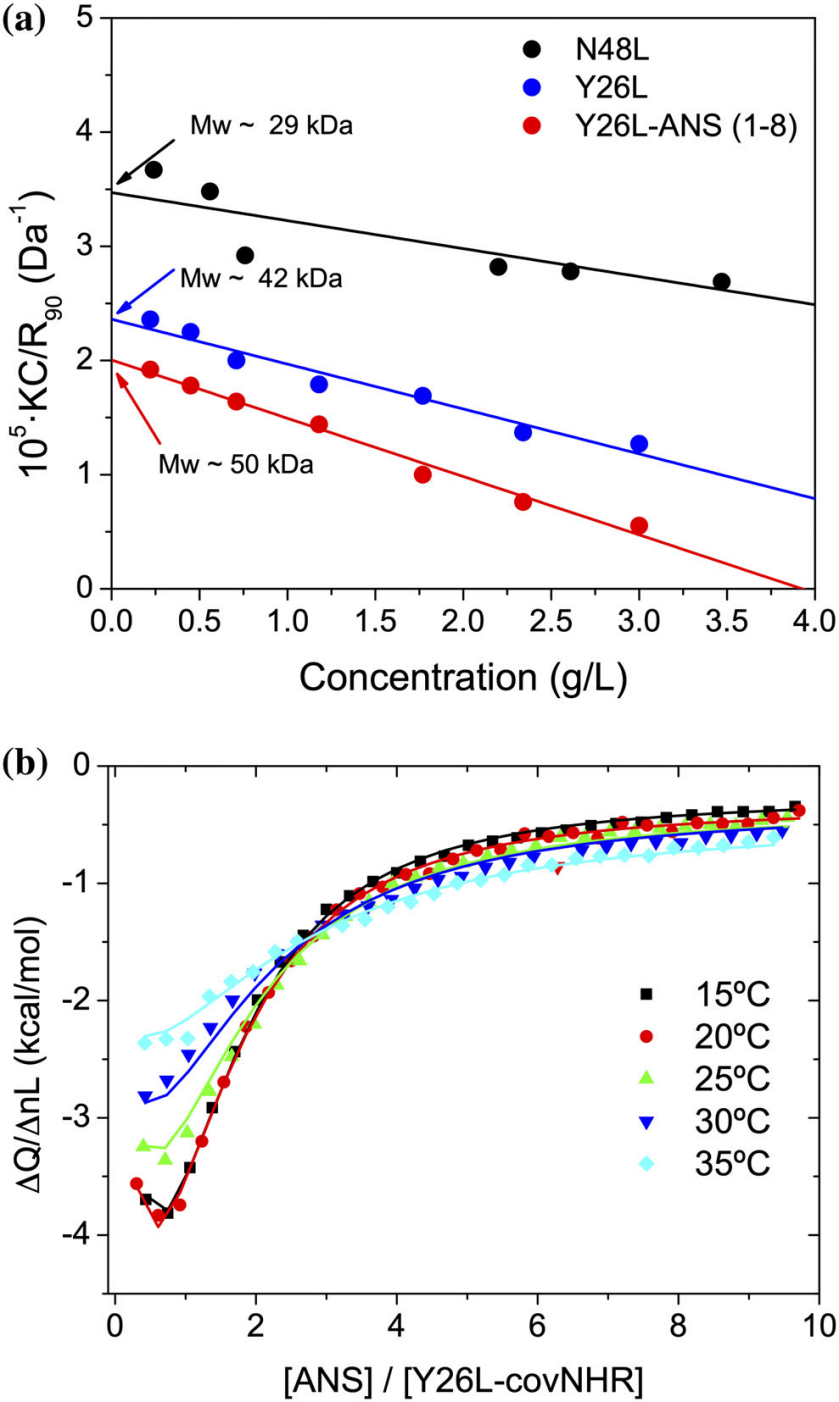
Binding of ANS to the Y26L‐chimera. (a) Debye plot derived from static scattering intensity measurements at different protein concentrations for N48L‐covNHR (black), Y26L‐covNHR (blue) and Y26L‐covNHR in the presence of 8‐fold molar excess of ANS (red). The intercepts of the linear dependences correspond to the weight‐averaged *M*_w_ of the molecules, as indicated. (b) Binding curves measured by ITC for the titration of Y26L‐covNHR with ANS at different temperatures. The symbols correspond to the experimental binding heats normalized per mole of added ANS in each injection. The lines represent the global fit using a model of ANS binding coupled to the chimera's dimerization (see Section 2.7 in the main text and Supplementary Appendix S2).

Given the relevance of intermolecular interactions mediated by the HP between the two Y26L‐covNHR monomers in the crystal state and their potential influence upon ANS binding, we further investigated the oligomerization state of the Y26L‐covNHR chimera in solution. The apparent hydrodynamic radius of Y26L‐covNHR measured by DLS at different concentrations showed a weak but significant increase at higher protein concentrations (from 2.8 to 3.2 nm) (Figure S7a), which was consistent with weak self‐association. In contrast, the hydrodynamic radius of the N48L‐covNHR chimera was independent of concentration and compatible with a monomer (Figure S7b). To confirm this, we performed static light scattering (SLS) measurements of the chimeras at different concentrations and constructed a Debye plot (Figure 6a). The results showed that, in solution, the Y26L‐covNHR chimera has a weight‐averaged *M*_w_ intermediate between the monomer and the dimer, suggesting a self‐association equilibrium, as also indicated by DLS measurements and the crystal structure. In contrast, the Debye plot obtained with N48L‐covNHR returned a *M*_w_ in good agreement with the monomer.

We further analyzed the Y26L‐ and N48L‐covNHR chimeric proteins by size‐exclusion chromatography (SEC) in a Superdex 75‐HR analytical column, previously calibrated with globular protein standards (Figure S8). The elution volumes of the chimeras corresponded to an apparent *M*_w_ of 33.1 and 33.7 kDa, respectively. These values are closer to the *M*_w_ of the monomers (23.6 kDa for Y26L and 26.7 kDa for N48L) than to those of the dimers. These discrepancies in apparent *M*_w_ relative to the expected values for a monomeric state could be explained by their rod‐like shape, which results in considerable increases in hydrodynamic radius compared to globular proteins of comparable size. These results indicated that, while the N48L is monomeric, the exposed HP of the Y26L chimera can mediate weak self‐association into dimers in solution, which become stabilized in the crystal structure. However, at low concentrations the Y26L‐covNHR monomer predominates in the dimerization equilibrium.

In the presence of an 8‐fold molar excess of ANS, the apparent hydrodynamic radius of Y26L‐covNHR measured by DLS further increased with the protein concentration (from 2.8 to 3.7 nm) (Figure S7c). Moreover, in the presence of an excess of ANS, the observed *M*_w_ of Y26L‐covNHR derived from the Debye plot was close to that of the dimer, indicating that ANS binding further stabilizes the dimeric state (Figure 6a). This is consistent with the observed interactions in the P_1_ crystal structure between ANS and groups of residues that belong to two different monomers of Y26L‐covNHR, that is, residues from the HP of one monomer and residues from the CHR of another.

### 2.7 Thermodynamics of ANS binding

To further characterize the binding of ANS to the Y26L‐covNHR chimera, we carried out ITC experiments at different temperatures. The ANS binding was exothermic, and the ITC curves showed a sigmoidal shape. However, the binding curves did not fit a model of independent and identical sites but could be fitted more accurately using a model of two sequential binding steps (Figure S9), as previously described for covNHR (Cano‐Muñoz et al. 2021). Nevertheless, none of these binding models fully explained the observed curves and did not account for the observed effects of ANS on the dimerization of the chimera. Therefore, we devised a model of ANS binding coupled to dimerization, supported by the experimental observations, in which we assumed that the free protein in solution undergoes a dimerization equilibrium, and then ANS binds independently to the HP of each molecule in the dimer (see Supplementary Appendix S2 for the mathematical development of the model). The model defined as fitting parameters the equilibrium dimerization constant of the chimera, *K*_D_, the microscopic binding constant of ANS to each HP in the dimer, *k*, as well as the binding enthalpies of each process, Δ*H*_D_ and Δ*H*_A_. We also added a final residual heat term as a fitting parameter to account for the dilution heats of ANS at the end of the titrations.

In individual fittings, the model reproduced the ITC curves with high accuracy (Figure S9), but the fitting parameters showed strong interdependencies, preventing their independent determination. Therefore, we carried out a global fitting of the complete set of ITC curves measured at different temperatures, introducing the heat capacity changes of dimerization, Δ*C*_pD_, and binding, Δ*C*_pA_, as additional fitting parameters that govern the temperature variation of the binding enthalpies. The fittings reproduced the effect of temperature on the shape and amplitude of the binding curves quite well (Figure 6b), given the strong constraints imposed by a common set of thermodynamic parameters (Table S7). In this global fitting, the heat capacity change of dimerization, Δ*C*_pD_, resulted in a value close to zero, so a very similar fit could be obtained by fixing this parameter to zero. The heat capacity of ANS binding was slightly positive, consistent with a compensation between the surface buried by ANS binding and the surface exposed by the displacement of the CHR N‐terminal segment from the HP. The equilibrium constant of dimerization of the chimera at 25°C is about 8 × 10^3^ M^−1^, which agrees with a weak dimerization, as observed in the light scattering experiments. At relatively low protein concentrations and in the absence of ANS, dimerization is disfavored, and the monomer predominates over the dimer (88% vs. 12% at 10 mM protein concentration). At higher protein concentrations or in the presence of ANS the equilibrium is shifted toward the dimeric state (Figure S10). The enthalpy of dimerization is positive (Δ*H*_D_ = +60 kJ mol^−1^), likely due to the need for partial lifting of the CHR N‐terminus to establish intermolecular contacts with the HP. Therefore, the dimerization of the chimera appears to be entropically favored by hydrophobic interactions and the liberation of hydration water. The binding affinity of ANS for each HP in the dimer is only moderate, with an estimated dissociation constant of 13 ± 1 μM, likely due to its small size and competition with interactions in the dimer. The ANS binding enthalpy is negative, indicating that ANS can establish stronger interactions at the HP than the displaced Tyr2 (Y638) side chain, due to the larger size of the naphthalene ring and the additional contacts made by the aniline moiety. Despite this competition between interactions, the HP shows a strong tendency to accommodate properly stacked, large hydrophobic groups.

## 3 DISCUSSION

In this work, we investigated the role of the HP in HIV‐1 inhibition using chimeric proteins mimicking the gp41 NHR region. The HP has been considered for decades an attractive target for different classes of inhibitors, including antibodies, small‐molecule compounds, as well as natural and artificial peptides, all of which bind the HP in the viral NHR region and interfere with membrane fusion (Eron et al. 2004; Miller et al. 2005; Qiu et al. 2019; Sia et al. 2002). Moreover, many constructs imitating an exposed NHR trimer targeting the viral CHR have been designed, including covalently linked NHR peptides (Bianchi et al. 2005; Louis et al. 2003; Wang et al. 2015), NHR trimers stabilized by a trimerization foldon (Chen et al. 2010a), single‐chain constructs forming an antiparallel NHR trimer (Crespillo et al. 2014), or 5‐helix constructs exposing an NHR groove (Root et al. 2001). All of them exhibited potent and broad HIV inhibitory activity, but they also systematically contained the exposed HP. However, it remains unclear whether the HP is a necessary and/or sufficient component of the design of NHR‐based protein constructs targeting the CHR region, and the mechanism by which the HP participates in inhibition.

In this work, we provide compelling evidence that exposing the HP alone in an NHR‐based inhibitor is insufficient to produce significant antiviral activity. The chimeric constructs studied here exhibited undetectable or very weak activity in HIV‐1 inhibition assays, irrespective of whether the HP is exposed (in the Y26L chimera) or occluded (in the N48L‐chimera). It could be argued that dimerization of Y26L‐covNHR chimera, involving interactions with the HP, could compromise its inhibitory activity. However, we have demonstrated that its dimerization in solution is weak and, under typical concentrations used in virus infection assays, the monomer largely predominates in the dimerization equilibrium. Moreover, the chimera binds the CHR peptide N25S with low micromolar affinity, demonstrating the HP exposure and its capacity to interact with the PBM.

This agrees with our previous study, in which we reported that a covNHR miniprotein (named covNHR‐C) mimicking the C‐terminal half of NHR was totally inactive to inhibit HIV‐1 infection, despite being fully monomeric, including the HP and the CTP, and binding the complementary CHR peptide N25S with micromolar affinity. However, despite its small size, covNHR‐C could not access the PBM in native Env (Jurado et al. 2020b). Conversely, the covNHR‐N miniproteins mimicking only the N‐terminal part of NHR and devoid of the HP, but harboring the NTP and MP pockets, could bind the C‐terminal half of CHR and exhibited potent nM inhibitory activity (Cano‐Muñoz et al. 2022; Jurado et al. 2020b; Polo‐Megías et al. 2025). This result demonstrated that the HP is not required for potent antiviral activity in these NHR‐based miniprotein inhibitors, whereas the MP and NTP pockets can be sufficient on their own. In pre‐fusion Env, the C‐terminal half of CHR forms a partially exposed α‐helix connected to the MPER by a polar flexible segment E^654^KNEQE^659^ (Pan et al. 2020). This polar motif is also highly preserved (Cano‐Muñoz et al. 2022) and, in the post‐fusion 6HB structure, establishes a water‐mediated hydrogen‐bond network with the NTP of the NHR region (Jurado et al. 2019). Based on the above evidence, it was proposed that the C‐terminal half of CHR might become exposed earlier than the PBM during gp41‐mediated fusion and therefore could constitute a key target of NHR‐based inhibitors (Jurado et al. 2020b).

Using a different approach, reverse hairpin CHR–NHR constructs were designed to stabilize the NHR trimers by partially covering the NHR groove with truncated CHR segments (Chu et al. 2015; Walsh et al. 2015). Only constructs that formed a stable NHR trimer and displayed an exposed HP exhibited potent inhibitory activity. However, the authors suggested that, in the virus‐cell fusion environment, membrane‐induced CHR–NHR dissociation and exposure of the remaining N‐terminal half of the NHR groove are also crucial for achieving nanomolar inhibition potency (He et al. 2024). Except for having a single‐chain topology, the chimeras described here share with these reversed hairpins an identical spatial arrangement between the CHR segment and the NHR groove.

In partial conflict with these results, some NHR trimeric constructs, such as IQN17, IZN17 or (CCIZN17)_3_, were designed to expose only the HP but not the other pockets of the NHR groove, and still exhibited potent inhibitory activity (Bianchi et al. 2005; Eckert and Kim 2001). Although the inhibition assays used in different studies may not be directly comparable to ours, it is unclear whether their potent inhibitory activity is attributable solely to their affinity for the PBM in gp41 CHR. The binding affinities of the HP for such a small CHR motif have been reported to be relatively weak, with K_d_ values in the low‐to‐medium micromolar range, even for conformationally constrained peptides or D‐peptides (Cole and Garsky 2001; Jurado et al. 2020a; Sia et al. 2002). This suggests that an alternative mechanism may be supporting the nanomolar‐to‐subnanomolar inhibitory activities reported for these constructs. The simultaneous exposure of three HP units in these constructs may enhance their effective binding affinity for the PBM in viral Env through an avidity mechanism. Other inhibition mechanisms could also play a role, such as the exchange of the NHR segment with the Env NHR trimer in prefusion intermediate (Bewley et al. 2002), or its embedment in the lipid membrane, which can disturb pore formation (Roche et al. 2014).

The results presented here do not imply that the HP would not play an active role in NHR‐based inhibitors, since the full covNHR protein, which displays the whole NHR groove, exhibits an increased activity compared to covNHR‐N, and a much higher affinity for isolated CHR peptides in solution (Jurado et al. 2019). It is possible that, in the viral Env context, after the inhibitor binds to the more accessible C‐terminal half of CHR by means of its MP and NTP pockets, a gp41 reorganization, possibly involving gp120 shedding, facilitates accessibility to the PBM, allowing HP binding via an avidity mechanism. This additional interaction may reduce the inhibitor's dissociation rate, but not increase its association rate, which sets a kinetic limit on the maximum activity of NHR‐based inhibitors (Kahle et al. 2009; Steger and Root 2006). Also, we and others have previously highlighted the crucial importance of the structural stability of the trimeric NHR coiled‐coil for HIV‐inhibition (Bianchi et al. 2005; Cano‐Muñoz et al. 2022; Dwyer et al. 2008; Polo‐Megías et al. 2025). The C‐terminal part of NHR, including the region that forms the HP, was found to be essential for promoting NHR trimerization. In contrast, the N‐terminal half appears to be intrinsically unstable and does not have a propensity to oligomerize, requiring interaction with CHR to self‐associate and form the 6HB structure (Dwyer et al. 2003).

In contrast to its role as part of an HIV inhibitor, the HP is crucial for gp41‐mediated membrane fusion. After receptor and co‐receptor binding, gp120 shedding and the concomitant release of the PBM allows its interaction with the HP. This initial 6HB nucleus may favor the propagation of the coiled‐coil 6HB, acting as a “foldon” to accelerate cooperatively a zippering‐like association of CHR with an otherwise conformationally flexible N‐terminal half of NHR. This would limit the exposure of critical NHR epitopes during gp41‐driven membrane fusion. We previously demonstrated long‐range allosteric communication between different pockets along the NHR groove (Jurado et al. 2020a).

This view agrees with the chief importance of discovering small molecules targeting the HP that could be developed as anti‐HIV drugs. However, this has been hampered by the lack of high‐resolution structural information of small‐molecule compounds bound to the HP. To the best of our knowledge, there are mainly computational docking studies, some assisted by NMR‐derived constraints (Balogh et al. 2009; Gochin et al. 2011), and only a crystal structure of a non‐peptidic inhibitor covalently linked to a CHR peptide has been previously reported (Zhou et al. 2000). Here, we report the first high‐resolution crystal structure of a free small‐molecule compound bound independently to the HP, without the need for external factors. ANS is an amphipathic compound with a naphthalene moiety similarly sized to the tryptophan indole group. Despite being a very well‐known and widely used fluorescent probe that binds weakly to exposed hydrophobic patches in proteins, ANS shows a moderate affinity for the HP in the Y26L‐covNHR chimera (*K*_d_ ≈13 μM), sufficient to allow for their co‐crystallization.

The high‐resolution crystal structure reveals interesting features that facilitate a better understanding of the interactions established at the HP and how a small‐molecule inhibitor could be rationally designed to achieve high affinity. In our complex structure, the naphthalene ring occupies the same position as the indole group of CHR Trp631 in the 6HB structure. The naphthalene ring makes hydrophobic contacts with residues lining the HP, but it is also stacked against the Leu5 side chain of the CHR segment from the opposite molecule in the asymmetric unit dimer. This Leu side chain occupies a position in the HP similar to that of the CHR Trp628 in the 6HB structure. The charged sulfonate group is oriented outward from the HP and establishes hydrogen bonds with the nearby amide groups of Leu5 and Ile6 in the CHR segment. The aniline moiety is laterally inserted between the side chains of Lys65 and Gln68 (K574 and Q577 in Env gp160 sequence). Surprisingly, the rest of the HP cavity is filled with water. All these features could be used as a guide to design new drug‐like derivatives or analogs of ANS imitating the above‐described interactions, leading to potential inhibitors targeting the HP.

Finally, an open question is whether the binding mode of ANS observed in the crystal structure is preserved in solution, and whether ANS can bind to the HP in the absence of the intermolecular contacts present in the chimera, given that the hydrophobic stacking described above involves elements contributed by both monomers of the asymmetric unit. The scattering measurements indicate the same ANS binding mode in solution, as ANS enhances the chimera's dimerization. Moreover, the ITC analysis indicates that ANS binds preferentially to the dimer, and the thermodynamic parameters are fully consistent with this interpretation. Our previous analysis of ANS binding to the covNHR protein showed that it also induced its dimerization (Cano‐Muñoz et al. 2021). Accordingly, given its small size, ANS is unlikely to establish sufficient interactions with the HP alone to allow tight binding; additional elements would be required to fill the HP. This is likely the reason we did not detect any anti‐HIV activity of ANS in TZM‐Bl assays (results not shown), since viral gp41 cannot reproduce a similar dimeric arrangement required for ANS to bind the HP. This does not diminish the value of the structural and thermodynamic information of ANS binding presented here. As shown in Figure 5, the HP is quite promiscuous in forming hydrophobic interactions with diverse motifs. However, the location and spatial orientation of the groups interacting with the HP are quite similar in all of them, suggesting a preferred geometry for hydrophobic/aromatic stacking that could be used to guide the design of larger ANS derivatives or homologs with higher affinity.

## 4 MATERIALS AND METHODS

### 4.1 Sample preparation

The DNA encoding the chimeras' sequences was synthesized and cloned into pET303 expression vectors (Thermo Fisher Scientific, Waltham, MA). The sequences (Table S1) included an N‐terminal methionine and a C‐terminal six‐histidine tag, with the sequence GGGGSHHHHHH. The proteins were overexpressed in *E. coli* BL21 (DE3) cells transformed with the plasmids. Protein purification was performed using nickel‐tag affinity (NTA) chromatography, followed by cation exchange chromatography, as previously described (Crespillo et al. 2014). The purity and identity of the proteins were assessed by SDS‐PAGE and mass spectrometry. Pure protein aliquots were stored frozen at −80°C. For biophysical characterization, the protein solutions were extensively dialyzed against the appropriate buffer and centrifuged at 14,000 rpm and 4°C for 30 min in a benchtop microcentrifuge before concentration measurement. Experiments were carried out in 50 mM sodium phosphate buffer pH 7.4 or in 50 mM glycine/HCl buffer pH 2.5.

The N25S peptide (Env residues Asn616‐Ser640) was synthesized by Genecust (Boynes, France) with a purity >95%, in N‐acetylated and C‐amidated form. Peptide solutions were freshly prepared by weighing the required amount of lyophilized peptide and dissolving it in the appropriate buffer, readjusting the pH if necessary. Then the sample was centrifuged at 14,000 rpm and 4°C for 30 min in a microcentrifuge to remove any insoluble material. Protein and peptide concentrations were measured by UV absorption at 280 nm, using extinction coefficients calculated from their respective amino acid sequences with the ExPasy ProtParam web server (https://web.expasy.org/protparam/) (Gasteiger et al. 2005).

8‐Anilino‐naphthalene‐1‐sulfonic acid (ANS) was purchased from Honeywell Fluka (Sigma‐Aldrich, St. Louis, MO). The concentration of ANS was quantified by UV–visible spectrophotometry using a molar extinction coefficient of 7800 M^−1^·cm^−1^ at 370 nm (Latypov et al. 2008).

### 4.2 Circular dichroism

Circular dichroism (CD) spectra were recorded in a Jasco J‐715 spectropolarimeter (Jasco, Tokyo, Japan) equipped with a Peltier thermostatic cell holder. Far‐UV CD spectra (260–200 nm) were measured at 25°C using a 1 mm path‐length quartz cuvette at a protein concentration of approximately 15 μM. Spectra were recorded at a scan rate of 100 nm/min, with a 1 nm step resolution, a 1 s response, and a 1 nm bandwidth. The resulting spectra were usually the average of five scans. CD spectra in the near‐UV wavelength range (450–250 nm) were acquired using a 0.5 cm path‐length cuvette with 10 averaged scans at a protein concentration of ≈40 μM. Each spectrum was baseline‐corrected by subtracting the buffer blank spectrum. Finally, the CD signal was normalized to molar ellipticity ([*θ*], in deg dmol^−1^ cm^2^).

### 4.3 Light scattering

The molecular size of the proteins was determined by dynamic light scattering (DLS) and static light scattering (SLS) measurements using a Malvern Zetasizer μV instrument (Worcestershire, UK). DLS data were measured at 25°C using a 1.5 mm quartz cuvette with an average number of 50 acquisitions and an acquisition time of 10 s. Immediately prior to measurements, the samples were centrifuged at 14,000 rpm and 4°C for 30 min to remove aggregates. Data were processed with the instrument's software to obtain the hydrodynamic radius distributions.

Static scattering intensities were also measured at different protein concentrations ranging from 0.2 to 3.5 mg/mL. The intensities were analyzed using the Debye plot as represented by Equation (1),

(1) K·cR90=1Mw+2A2c,

valid for particles significantly smaller than the wavelength of the incident radiation, where *K* is an optical constant of the instrument, *c* is the particle mass concentration, *R*_90_ is the Rayleigh ratio of scattered to incident light intensity, *M*_w_ is the weight‐averaged molar mass, and *A*_2_ is the 2nd virial coefficient that is representative of inter‐particle interaction strength. *M*_w_ can be determined from the plot's intercept.

### 4.4 Size‐exclusion chromatography

The oligomerization state of the Y26L‐covNHR and N48L‐covNHR chimeras was further characterized by SEC using a Superdex 75 10/300 GL column connected to an ÄKTAprime plus FPLC system (GE Healthcare Bio‐Sciences AB, Sweden) operating at a flow rate of 1 mL/min. The elution buffer was 50 mM sodium phosphate pH 7.4, 100 mM NaCl. 100 μL protein samples were injected at about 50 μM protein concentration. The column was calibrated with several globular protein standards of different molecular weights under the same experimental conditions.

### 4.5 Differential scanning calorimetry

Differential scanning calorimetry (DSC) experiments were carried out in a Malvern PEAQ‐DSC microcalorimeter equipped with an autosampler (Malvern Panalytical, Malvern, UK). Scans were typically performed from 5 to 125°C, at a scan rate of 120°C h^−1^ and a protein concentration of about 30 μM, unless stated specifically. Instrumental baselines were recorded before each experiment with both cells filled with buffer and subtracted from the experimental thermograms of the protein samples. Consecutive reheating runs were performed to assess the reversibility of thermal denaturation. The excess heat capacity (Δ*C*_p_) relative to the buffer was calculated from the experimental DSC thermograms using Origin software (OriginLab, Northampton, MA) and normalized per mole of protein.

### 4.6 Isothermal titration calorimetry

Isothermal titration calorimetry (ITC) measurements were carried out using a Microcal VP‐ITC microcalorimeter (Malvern Instruments, Worcestershire, UK). For protein‐peptide binding analysis, a 10 μM protein solution was titrated with 30 injections of 10 μL of a 0.14 mM peptide solution at 480 s intervals. The experiments were carried out in 50 mM sodium phosphate buffer, pH 7.4. For ANS binding analysis, 25 μM samples of Y26L‐covNHR chimera were titrated with 30 injections of 10 μL of ANS at about 1.2 mM. Experiments were carried out at five different temperatures between 15 and 35°C. The experimental thermograms were baseline corrected, and the peaks were integrated to determine the heats of each ligand injection. Each heat was then normalized per mole of added ligand. Binding isotherms were analyzed with Origin 8.6 (Originlab, Northampton, MA) using different binding models as described in section 2 and Supplementary Appendix S2.

### 4.7 Protein crystallization

Screening for initial crystallization conditions was performed using the sitting‐drop vapor‐diffusion method with the commercially available crystal screening kits, Proplex and Structure Screen 1 and 2, from Molecular Dimensions (Suffolk, UK). Concentrated solutions of each chimera were prepared in 10 mM Tris, pH 8, at 8 mg/mL. Droplets consisting of 2 μL protein solution and 2 μL reservoir solution were equilibrated at 25°C against 200 μL reservoir solution in 48‐well MRC Maxi Optimization plates (Cambridge, UK). Several favorable conditions were initially identified for the Y26L‐covNHR chimera and were optimized to obtain improved crystals. The best diffracting crystals of Y26L‐covNHR were obtained using the sitting‐drop setup, with 3 μL protein solution droplets at 8 mg/mL and 1 μL reservoir solution, equilibrated against 200 μL reservoir solution composed of 0.2M ammonium sulfate, 30% w/v PEG 8000, and 0.1M MES, pH 6.5.

For co‐crystallization of Y26L‐covNHR and ANS, the stock protein solution was dialyzed in 10 mM Tris–HCl buffer, pH 8, and concentrated to 8 mg/mL. Then, ANS was added to reach an 8:1 molar excess. Crystals of the Y26L‐covNHR/ANS complex were obtained in the same conditions as the free form of the protein.

### 4.8 Data collection, structure solution, and refinement

Before data collection, the protein crystals were flash‐cooled in liquid nitrogen. Data sets were collected at 100 K at the beamline ID23‐2 at the ESRF (Grenoble, France) and BL13‐XALOC at the ALBA synchrotron (Barcelona, Spain) (Juanhuix et al. 2014; McCarthy et al. 2018). Diffraction data were indexed and integrated with the AutoPROC toolbox (Vonrhein et al. 2011). Data scaling was performed using the program Aimless (Evans 2011) from the CCP4 suite (Agirre et al. 2023). Data collection statistics are collected in Table S4. Solution and refinement of the structures were performed using the PHENIX suite (Adams et al. 2010). Molecular‐replacement phasing using PHASER (Bunkóczi et al. 2013) was performed with the coordinates of the crystallographic structure of the single‐chain protein mimetic of the gp41 NHR trimer in complex with the synthetic CHR peptide C34 (PDB entry 6R2G; Jurado et al. 2019). Manual model‐building was performed using COOT (Emsley et al. 2010; Emsley and Cowtan 2004). Refinement was performed using phenix.refine in PHENIX (Afonine et al. 2012). Quality of the structures was checked using Molprobity (Chen et al. 2010b) and Procheck (Laskowski 2001). Structural refinement statistics are collected in Table S4. The structure coordinates were deposited at the Protein Data Bank (PDB) under the accession codes 9FED and 9SJQ for the free form of Y26L‐covNHR, and 9SJP for the Y26L‐covNHRL/ANS complex.

### 4.9 Virus inhibition assays

Virus inhibition was analyzed using either the conventional TZM‐Bl neutralization assay or a PBMC‐based assay with primary virus stocks (Heyndrickx et al. 2012; Sarzotti‐Kelsoe et al. 2014). The viruses and plasmids were obtained through the NIH HIV Reagent Program, Division of AIDS, NIAID, NIH. Pseudoviruses SF162 and MW965.26 were produced by co‐transfection with SF162 or MW965.26 Env plasmids and HIV pSG3 delta Env backbone. Primary isolates of SF162 and 92RW009 strains were produced on PBMC. Virus stocks were titrated to obtain about 20,000 RLU for the TZM‐Bl assay and 5–10% p24‐positive infected cells for the PBMC assay. TZM‐Bl based assay was performed with two pseudoviruses (SF162 of clade B and MW965.26 of clade C) and a primary isolate 92RW009 (clade A). PBMC‐based assay was performed using the SF162 and 92RW009 primary isolates (virus stocks produced in PBMC). Three days after infection, PBMC were stained for the detection of intracellular p24 to measure the number of infected cells. The inhibitory concentration 50 (IC_50_) was calculated as the concentration of the molecule required to reduce virus growth by 50%. To identify potential inhibitor toxicity, staining was performed using LIVE/DEAD™ Fixable Violet Dead Cell Stain (Molecular Probes) before intracellular p24 staining.

## AUTHOR CONTRIBUTIONS

**Daniel Polo‐Megías:** Conceptualization; investigation; formal analysis; data curation; supervision; writing – review and editing. **Mario Cano‐Muñoz:** Conceptualization; investigation; supervision; writing – review and editing. **Laura Sánchez‐Martínez:** Investigation. **Sara Lestani:** Investigation. **Christiane Moog:** Investigation; supervision; project administration; funding acquisition; supervision. **Thomas Decoville:** Investigation. **M. Carmen Salinas‐García:** Investigation. **José A. Gavira:** Investigation; data curation. A**na Cámara‐Artigas:** Investigation; formal analysis; writing – review and editing. **Francisco Conejero‐Lara:** Conceptualization; data curation; formal analysis; methodology; funding acquisition; project administration; software; supervision; visualization; writing – original draft.

## CONFLICT OF INTEREST STATEMENT

The authors declare no conflicts of interest.

## Supporting information


**Table S1.** Amino acid sequences of the gp160 CHR and NHR regions and the three CHR‐covNHR quimeras studied in this work.
**Figure S1.** Hydrodynamic radius distributions of the chimeras measured by dynamic light scattering (DLS).
**Table S2.** Biophysical properties of the covNHR protein in comparison with the CHR‐covNHR chimeras.
**Table S3.** Thermodynamic parameters of binding of the N25S peptide to the Y26L‐covNHR chimera measured by ITC using a binding model of N independent and equivalent sites.
**Table S4.** Data collection and refinement statistics of the crystallographic structures of Y26‐covNHR.
**Figure S2.** Root mean squared deviations between equivalent Ca atoms of Y26LcovNHR and the covNHR:C34 complex.
**Appendix S1.** Analysis of interactions in the crystal structures of Y26L‐covNHR.
**Figure S3.** Intermolecular arrangement between simmetry‐related Y26L‐covNHR monomers in the C2_1_ crystal polymorph.
**Table S5.** Intermonomer interaction analysis between Y26L‐covNHR monomers in the C2_1_ crystal polymorph.
**Figure S4.** Intermolecular arrangement between Y26L‐covNHR chains A and B in the P_1_ crystal polymorph and comparison with the C21 polymorph.
**Figure S5.** Polder maps for ligand ANS in chain A (left) and B (right).
**Figure S6.** Details of the interactions between ANS and the Y26L‐covNHR residues in the P1 crystal.
**Table S6.** Analysis of the interactions between ANS and Y26L‐covNHR monomers in the P1 crystal polymorph.
**Figure S7.** DLS analysis of the molecular size of the chimeras in solution.
**Figure S8.** Size‐exclusion chromatography (SEC) analysis of the chimeras.
**Figure S9.** ITC titration of Y26L‐covNHR with ANS.
**Appendix S2.** Mathematical development of the model of ligand binding coupled to dimerization.
**Table S7.** Thermodynamic parameters of binding of ANS to the Y26L‐covNHR chimera measured by ITC using a model of binding coupled to dimerization.).
**Figure S10.** Fraction of Y26L‐covNHR in the dimeric state as a function of the ANS/protein molar ratio.

## Data Availability

The data that support the findings of this study are available from the corresponding author upon reasonable request.
