# Supplementary material for: Investigating the role of a conserved hydrophobic pocket of gp41 in the anti‐HIV activity of fusion inhibitors
Source: Protein Sci. 2026 May 4;35(6):e70593. doi: 10.1002/pro.70593 (PMC13137300; doi:10.1002/pro.70593)
Supplement: Supplementary file 1 — Table S1. Amino acid sequences of the gp160 CHR and NHR regions and the three CHR‐covNHR quimeras studied in this work. Figure S1. Hydrodynamic radius distributions of the chimeras measured by dynamic light scattering (DLS). Table S2. Biophysical properties of the covNHR protein in comparison with the CHR‐covNHR chimeras. Table S3. Thermodynamic parameters of binding of the N25S peptide to the Y26L‐covNHR chimera measured by ITC using a binding model of N independent and equivalent sites. Table S4. Data collection and refinement statistics of the crystallographic structures of Y26‐covNHR. Figure S2. Root mean squared deviations between equivalent Ca atoms of Y26LcovNHR and the covNHR:C34 complex. Appendix S1. Analysis of interactions in the crystal structures of Y26L‐covNHR. Figure S3. Intermolecular arrangement between simmetry‐related Y26L‐covNHR monomers in the C21 crystal polymorph. Table S5. Intermonomer interaction analysis between Y26L‐covNHR monomers in the C21 crystal polymorph. Figure S4. Intermolecular arrangement between Y26L‐covNHR chains A and B in the P1 crystal polymorph and comparison with the C21 polymorph. Figure S5. Polder maps for ligand ANS in chain A (left) and B (right). Figure S6. Details of the interactions between ANS and the Y26L‐covNHR residues in the P1 crystal. Table S6. Analysis of the interactions between ANS and Y26L‐covNHR monomers in the P1 crystal polymorph. Figure S7. DLS analysis of the molecular size of the chimeras in solution. Figure S8. Size‐exclusion chromatography (SEC) analysis of the chimeras. Figure S9. ITC titration of Y26L‐covNHR with ANS. Appendix S2. Mathematical development of the model of ligand binding coupled to dimerization. Table S7. Thermodynamic parameters of binding of ANS to the Y26L‐covNHR chimera measured by ITC using a model of binding coupled to dimerization.). Figure S10. Fraction of Y26L‐covNHR in the dimeric state as a function of the ANS/protein molar ratio. [file PRO-35-e70593-s001.pdf]

Supplementary Information to:

Investigating the role of a conserved hydrophobic pocket of gp41 in the anti-HIV activity of fusion inhibitors.

Daniel Polo-Megías[1], Mario Cano-Muñoz[1], Laura Sánchez-Martínez[1], Sara Lestani[1], Christiane Moog[2,5], Thomas Decoville[2], M. Carmen Salinas-García[1], José A. Gavira[3], Ana Cámara-Artigas[4] & Francisco Conejero-Lara[1,*]

[1] Departamento de Química Física, Instituto de Biotecnología y Unidad de Excelencia de Química Aplicada a Biomedicina y Medioambiente (UEQ), Facultad de Ciencias, Universidad de Granada, 18071 Granada, Spain

[2] Laboratoire d'ImmunoRhumatologie Moléculaire, Institut National de la Santé et de la Recherche Médicale (INSERM) UMR_S 1109, Institut Thématique Interdisciplinaire (ITI) de Médecine de Précision de Strasbourg, Transplantex NG, Faculté de Médecine, Fédération Hospitalo-Universitaire OMICARE, Fédération de Médecine Translationnelle de Strasbourg (FMTS), Université de Strasbourg, F-67000 Strasbourg, France

[3] Laboratorio de Estudios Cristalográficos, IACT-CSIC, Armilla, 18100 Granada, Spain

[4] Department of Chemistry and Physics, University of Almería, Agrifood Campus of International Excellence (ceiA3), Research Center for Mediterranean Intensive Agrosystems and Agri-Food Biotechnology (CIAIMBITAL), Carretera de Sacramento s/n, Almería, 04120, Spain

[5] Vaccine Research Institute (VRI), F-94000 Créteil, France

Table of contents

Table S1. Amino acid sequences of the CHR and NHR regions of gp160 and the three CHR-covNHR quimeras studied in this work *3*

Figure S1. Hydrodynamic radius distributions of the chimeras measured by dynamic light scattering (DLS). *4*

Table S2. Biophysical properties of the covNHR protein in comparison with the CHR-covNHR chimeras *5*

Table S3. Thermodynamic parameters of binding of the N25S peptide to the Y26L-covNHR chimera *5*

Table S4. Data collection and refinement statistics of the crystallographic structures of Y26-covNHR *6*

Figure S2. Root mean squared deviations between equivalent Cα atoms of Y26L-covNHR and the covNHR:C34 complex. *7*

Appendix S1. Analysis of interactions in the crystal structures of Y26L-covNHR *8*

Figure S3. Intermolecular arrangement between simmetry-related Y26L-covNHR monomers in the $C2_1$ crystal polymorph. *10*

Table S5. Intermonomer interaction analysis between Y26L-covNHR monomers in the $C2_1$ crystal polymorph. *11*

Figure S4. Intermolecular arrangement between Y26L-covNHR chains A and B in the P_1 crystal polymorph and comparison with the $C2_1$ polymorph. *13*

Figure S5. Polder maps for ligand ANS in chain A (left) and B (right). *14*

Figure S6. Details of the interactions between ANS and the Y26L-covNHR residues in the P_1 crystal. *14*

Table S6. Analysis of the interactions between ANS and Y26L-covNHR monomers in the P_1 crystal polymorph *15*

Figure S7. DLS analysis of the molecular size of the chimeras in solution. *16*

Figure S8. Size-exclusion chromatography (SEC) analysis of the chimeras. *17*

Figure S9. ITC titration of Y26L-covNHR with ANS. *18*

Appendix S2. Mathematical development of the model of ligand binding coupled to dimerization *19*

Table S7. Thermodynamic parameters of binding of ANS to the Y26L-covNHR chimera measured by ITC *22*

Figure S10. Fraction of Y26L-covNHR in the dimeric state as a function of the ANS/protein molar ratio. *22*

Supplementary references *23*

Table S1. Amino acid sequences of the CHR and NHR regions of gp160 and the three CHR-covNHR quimeras studied in this work. [a]

Fragment	Amino acid sequence
	**gp160**
gp160-NHR	^{541}ARQLLSGIVQQQNNLLRAIEAQQHLLQLTVWGIKQLQARILAVERYLKDQQL^{592}
gp160-CHR	^{616}NKSLEQIWNNMTWMEWDREINNYTSLIHSLIEESQNQQEKNEQELLEL^{663}
	**CHR fragment** [b]
Y26L	MYTSLIHSLIEESQNQQEKNEQELLEL
M38L	MTWMEWDREINNYTSLIHSLIEESQNQQEKNEQELLEL
N48L	MNKSLEQIWNNMTWMEWDREINNYTSLIHSLIEESQNQQEKNEQELLEL
	**covNHR fragment** [c]
Loop 1	GEGE
NHR 1	ARQELSGIVQKQNNLLRQIEAQQHLLQLTVSKIKQLQARILAVERYLKDQQL
Loop 2	GKGNQ
NHR 2	PQQDKLYREVALIRAQLQKIESETLQLLHQQAEIERELNNQEQEIGSLKQR
Loop 3	GLIDG
NHR 3	PLLSGIDQQQNNLKRAIEAQKHLLQLTVWGIKQLQARILAVERYLKDQQL

[a] The three proteins contain a C-terminal histidine tag with sequence GGGGSHHHHHH.

[b] The chimeras differ in the CHR fragment N-terminally fused to the covNHR fragment.

[c] The same in the three chimeras.

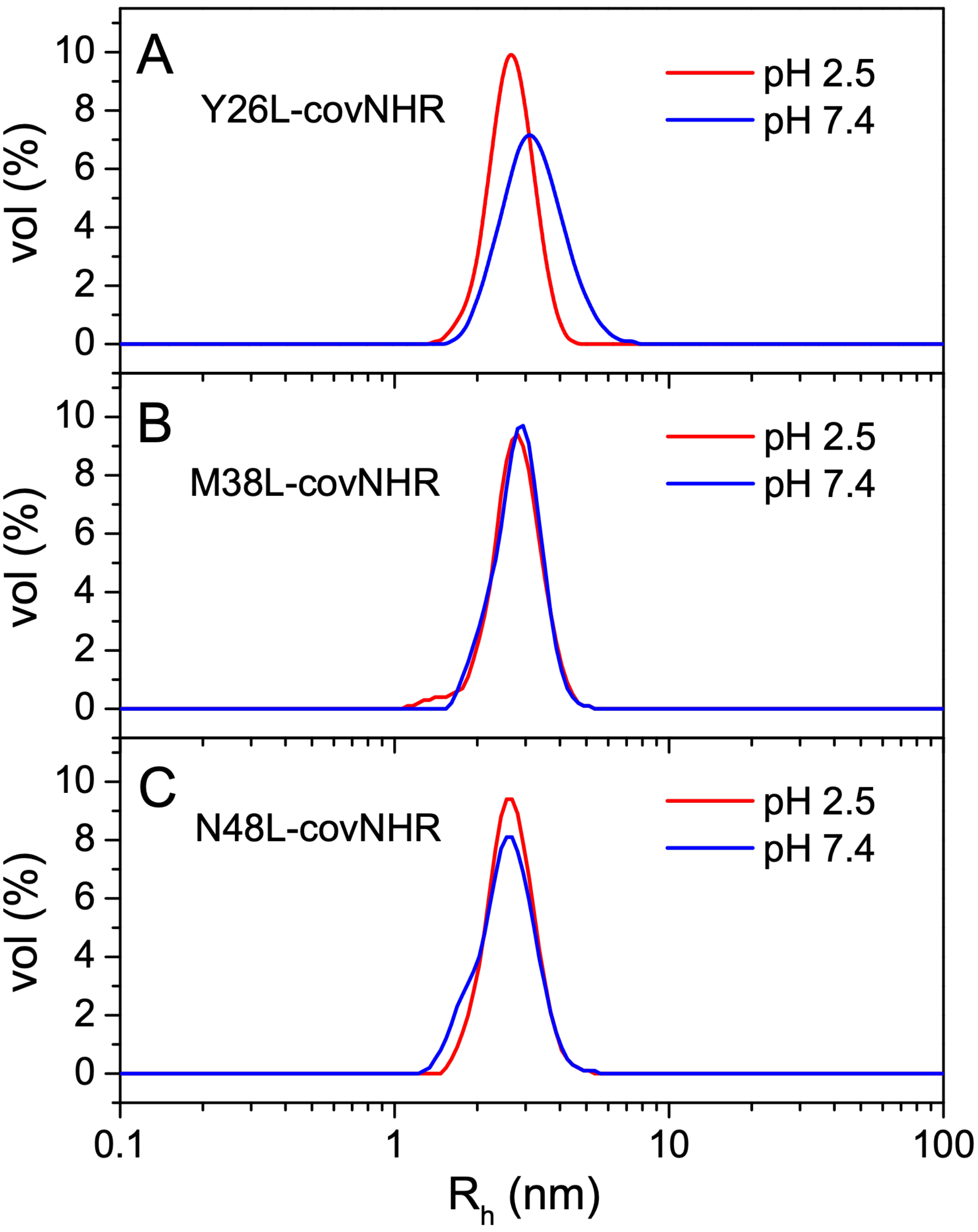

Figure S1. Hydrodynamic radius distributions of the chimeras measured by dynamic light scattering (DLS). Experiments were carried out at 25°C in 50 mM sodium phosphate buffer pH 7.4, and 50 mM glycine/HCl buffer pH 2.5. Protein concentration was about 15 μM in these experiments.

Table S2. Biophysical properties of the covNHR protein in comparison with the CHR-covNHR chimeras.

Protein	R_h [(a)] (nm)	% α-helix[(b)]	Residues in α-helix / total	T_m [(c)] (°C)	ΔH_m [(d)] (kJ mol^{-1})
			pH 2.5 (50 mM glycine/HCl)		
covNHR[(e)]	2.7	84.9	149 / 176	89.8	501
Y26L-covNHR	2.7	82.3	169 / 205	93.9	867
M38L-covNHR	2.8	84.3	182 / 216	(103.7) 106.5	1108
N48L-covNHR	2.6	77.3	175 / 227	106.5	1163
			pH 7.4 (50 mM sodium phosphate)		
covNHR[(d)]	2.8	80.4	141 / 176	108.3	369
Y26L-covNHR	3.1	78.0	160 / 205	108.4	561
M38L-covNHR	2.9	83.1	179 / 216	117.7	778
N48L-covNHR	2.6	81.7	185 / 227	119.8	870

[(a)] Hydrodynamic radius measured by dynamic light scattering at 25°C.
[(b)] Estimated from the mean-residue ellipticity at 222 nm. [1]
[(c)] Unfolding temperature measured from the maximum of the unfolding peaks measured by differential scanning calorimetry (DSC).
[(d)] Unfolding enthalpy estimated as the area under the DSC peaks.
[(e)] Data taken from [2].

Table S3. Thermodynamic parameters of binding of the N25S peptide to the Y26L-covNHR chimera measured by ITC using a binding model of N independent and equivalent sites.

Ligand	N[(a)]	K_b (M^{-1})[(b)]	K_d (µM)[(c)]	ΔH_b (kJ·mol^{-1})[(d)]
N25S	1.25	$(3.0 \pm 0.7) \times 10^5$	3.3 ± 0.8	-63 ± 6

[(a)]Apparent binding stoichiometry
[(b)]Binding equilibrium constant
[(c)]Dissociation constant
[(d)]Binding enthalpy

Table S4. Data collection and refinement statistics of the crystallographic structures of Y26-covNHR

	Y26L-VQ-ANS	Y26L-VQ	Y26L-VQ
PDB code	9SJP	9SJQ	9FED
Wavelength (Å)	0.98	0.98	0.87
Resolution range	19.47- 1.15 (1.16-1.15)	19.93 - 1.50 (1.53-1.50)	18.75- 2.30 (2.38 - 2.30)
Space group	P 1	C 2 1	C 2 1
Unit cell (Å)	47.5 53.1 58 70.02 69.93 71.65	53.75 79.71 62.90 90 94.20 90	50.36 79.36 56.84 90 100.34 90
Total reflections	274602 (9970)	117626 (5470)	30170 (2988)
Unique reflections	154137 (6335)	41887 (2032)	9644 (954)
Multiplicity	1.8 (1.6)	2.8 (2.7)	3.1 (3.1)
Completeness (%)	88.9 (73.6)	99.1 (98.8)	98.4 (99.0)
Mean I/sigma(I)	12.5 (1.7)	8.1 (1.2)	6.1 (1.8)
Wilson B-factor	13.7	22.1	31.2
R-merge	0.031 (0.518)	0.047 (0.509)	0.078 (0.523)
R-meas	0.043 (0.733)	0.066 (0.705)	0.110 (0.731)
R-pim	0.031 (0.518)	0.046 (0.487)	0.077 (0.509)
CC1/2	0.99 (0.84)	0.99 (0.94)	0.99 (0.68)
Reflections used in refinement	154114 (4029)	41833 (4133)	9641 (967)
Reflections used for R-free	7840 (205)	2127 (215)	438 (39)
R-work	0.15 (0.24)	0.18 (0.27)	0.22 (0.28)
R-free	0.17 (0.27)	0.21 (0.32)	0.27 (0.35)
Number of non-hydrogen atoms	3744	1748	1492
macromolecules	3210	1590	1463
ligands	64	15	0
solvent	470	143	29
Protein residues	387	193	184
RMS(bonds)	0.009	0.007	0.002
RMS(angles)	1.03	0.90	0.44
Ramachandran favored (%)	99.74	97.38	98.89
Ramachandran allowed (%)	0.26	2.62	1.11
Ramachandran outliers (%)	0.00	0.00	0.00
Rotamer outliers (%)	0.28	0.57	0.00
Clashscore	1.36	1.85	2.72
Average B-factor	22.74	40.17	49.99
macromolecules	21.37	39.41	50.13
ligands	34.39	89.67	-
solvent	30.53	43.42	43.05
Number of TLS groups	-	-	3

Statistics for the highest-resolution shell are shown in parentheses.

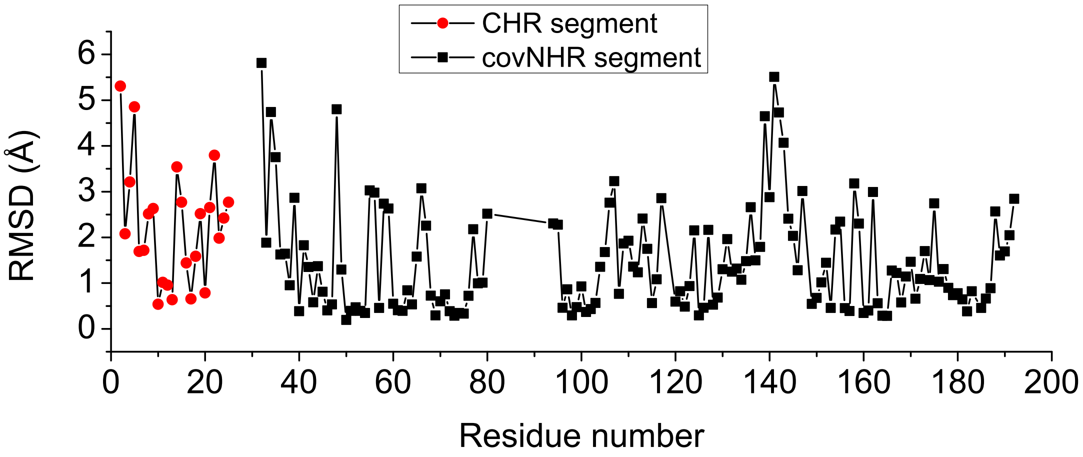

Figure S2. Root mean squared deviations between equivalent Cα atoms of Y26L-covNHR and the covNHR:C34 complex. Gaps correspond to unresolved or non-equivalent regions.

Appendix S1. Analysis of interactions in the crystal structures of Y26L-covNHR

Crystallographic structures of the Y26L-covNHR chimera in free form ($C2_1$ crystal polymorph) and bound to ANS (P_1 polymorph) were analyzed using YASARA structure software [3].

A) Crystal polymorph belonging to the $C2_1$ group

Two chimera monomers in the $C2_1$ crystal polymorph associate as a symmetric dimer with a contact surface area of 613 Å^2 per monomer, of which 95% corresponds to hydrophobic surface. The intermolecular contacts involve mainly residues from the segment Tyr2-Leu9 (Y638-L645 in gp160 numbering) of the CHR N-terminus of one monomer and residues that constitute the hydrophobic pocket of another (see Table S5), but there are also contacts between residues from the third NHR helices of each monomer. The dimer interface is also stabilized by six intermolecular hydrogen bonds and three pi-stacking interactions. There are also five sulphate ions laterally associated near the dimer interface establishing several electrostatic interactions and hydrogen bonds with polar sidechains.

B) Crystal polymorph belonging to the P_1 group

The two chimera monomers in the crystal polymorph P_1, which contain ANS bound to the HP, show an arrangement in the dimer that is very similar to that observed in the $C2_1$ crystal (Figure S4A). However, the presence of bound ANS breaks the $C2_1$ symmetry and produces a significant conformational rearrangement. Besides of the ANS molecules bound to the HP of each monomer, an aniline moiety is observed bound to a small pocket of monomer 2, producing a significant distortion at the nearest loop. This aniline may be a result of ANS degradation, since ANS is known to be a photosensitive compound. The asymmetric unit shows only three sulphate ions bound to the dimer. The global RMSD between the C alpha atoms of the dimers is 1.2 Å, and most deviations are concentrated in and near the loops (Figure S4B).

The ANS bound to the HP shows a partial occupancy of 0.49 in both monomers, inducing two alternate conformations for residues Tyr2 (Y638), Thr3 (T639) and Ser4 (S640) at the CHR N-terminus, which occupy the HP in the absence of bound ANS (with occupancies ranging between 0.35 and 0.64) or become displaced from the HP. In the latter case Tyr2 does not show visible electron density.

We analyzed the interactions of bound ANS with the two monomers of the chimera in the crystal. Contact surface area of ANS with residues of the HP in monomer 1 is 87 Å^2, mostly hydrophobic (86%). ANS also buries 63 Å^2 in contacts with the N-terminus of the CHR segment of monomer 2. The interactions between ANS and the chimeras are listed in Table S6. ANS establishes extensive hydrophobic contacts with residues of the HP, displacing Tyr2 from the HP. Both the number of contacts and the interaction strength of ANS are greater than those of Tyr2, due to the larger size of the naphthalene ring and the participation of the aniline ring, which makes contacts with the aliphatic part of Lys65 (K574) and Gln68 (Q577) sidechains. Moreover, the naphthalene ring of ANS makes a π-π stacking with the indole ring of Trp173 (W571), resembling the binding mode of Trp631 in the 6-helix bundle gp41 structure. This binding mode is also favored by hydrophobic interactions with Leu5 (L641) from the CHR segment of the second monomer, which occupies a similar position as Trp628 side chain in the 6-HB gp41

conformation. Besides, the sulphonate group of ANS makes two hydrogen bonds with the main chain amide groups of Leu5 (L641) and Ile6 (I642), capping the CHR helix and thus helping to stabilize the binding mode. All these interactions explain the favorable thermodynamics of binding of ANS to the chimera dimer observed in solution.

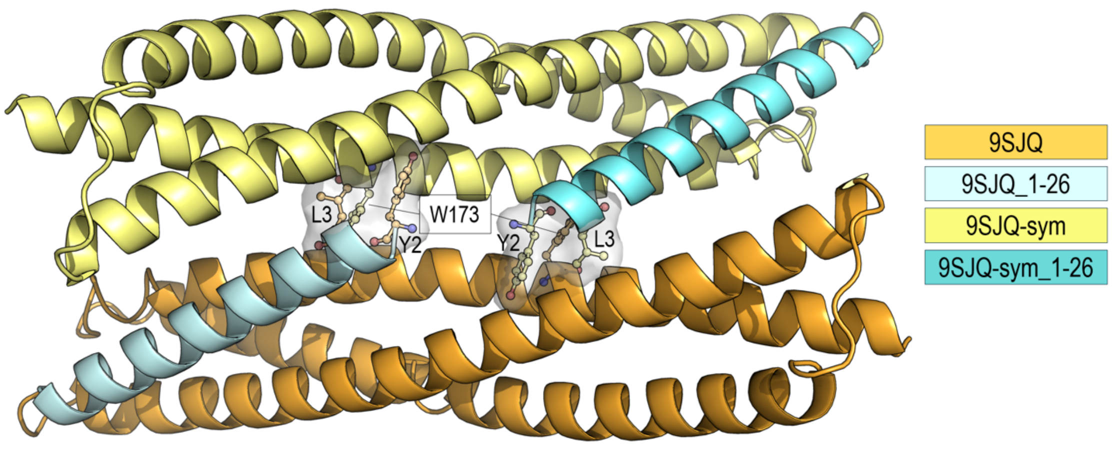

Figure S3. Intermolecular arrangement between simmetry-related Y26L-covNHR monomers in the C2$_1$ crystal polymorph. Ribbon representation of the two associated monomers of Y26L-covNHR in the C2$_1$ crystal. The covNHR segments of each monomer are colored in orange and yellow respectively, and the CHR segments in pale and intense cyan. The contacts between Tyr2 (Y638) and Leu5 (L641) side chains, from the CHR of one monomer, and Trp173 (W571) side chain, from the HP of the opposite monomer, are highlighted.

Table S5. Intermonomer interaction analysis between Y26L-covNHR monomers in the C2₁ crystal polymorph. Residue equivalence with gp160 sequence numbering is indicated with parentheses.

Hydrophobic interactions (cutoff distance: 5 Å)

Residue from Monomer 1 (gp160 equivalent)	Residue from Monomer 2 (gp160 equivalent)	Number of contacts [(a)]	Interaction Strength [(b)]
TYR 2 (Y638)	VAL 61 (V570)	1	0.884
	ILE 64 (I573)	1	0.277
	LYS 65 (K574)	3	3.12
	LEU 170 (L568)	3	2.468
	THR 171 (T569)	2	2.266
	TRP 173 (W571)	2	1.37
SER 4 (S640)	GLN 68 (Q577)	1	0.525
LEU 5 (L641)	ILE 64 (I573)	1	0.656
	GLN 68 (Q577)	3	2.743
	TRP 173 (W571)	3	2.625
	GLY 174 (G572)	1	0.734
	GLN 177 (Q575)	2	1.735
	LEU 178 (L576)	3	1.889
ILE 6 (I642)	TRP 173 (W571)	1	0.4
SER 8 (S644)	ARG 181 (R579)	1	0.849
LEU 9 (L645)	TRP 173 (W571)	1	0.762
VAL 61 (V570)	TYR 2 (Y638)	1	0.912
ILE 64 (I573)	TYR 2 (Y638)	1	0.283
	LEU 5 (L641)	1	0.675
LYS 65 (K574)	TYR 2 (Y638)	4	3.048
GLN 68 (Q577)	SER 4 (S640)	1	0.516
	LEU 5 (L641)	3	2.687
GLU 162 (E560)	TRP 173 (W571)	1	0.898
ALA 163 (A561)	LEU 170 (L568)	1	0.946
	TRP 173 (W571)	2	1.13
HIS 166 (H564)	HIS 166 (H564)	1	0.707
	GLN 169 (Q567)	1	0.796
	LEU 170 (L568)	4	3.381
LEU 167 (L565)	LEU 170 (L568)	3	2.307
GLN 169 (Q567)	HIS 166 (H564)	1	0.795
LEU 170 (L568)	TYR 2 (Y638)	3	2.354
	ALA 163 (A561)	1	0.944
	HIS 166 (H564)	4	3.606
	LEU 167 (L565)	2	2.279
	LEU 170 (L568)	1	0.662

TRP 173 (W571)	TYR 2 (Y638)	2	2.297
	LEU 5 (L641)	3	2.585
	ILE 6 (I642)	1	0.366
	LEU 9 (L645)	2	1.409
	GLU 162 (E560)	1	0.883
	ALA 163 (A561)	2	1.161
GLY 174 (G572)	TYR 2 (Y638)	2	1.385
	LEU 5 (L641)	1	0.635
GLN 177 (Q575)	LEU 5 (L641)	2	1.736
LEU 178 (L576)	LEU 5 (L641)	3	1.9
ARG 181 (R579)	SER 8 (S644)	1	0.856

π-π interactions (cutoff distance: 5 Å)

Residue from Monomer 1	Residue from Monomer 2	Number of contacts (c)	Interaction Strength (d)
TYR 2 (Y638)	TRP 173 (W571)	1	0.08
HIS 166 (H564)	HIS 166 (H564)	2	0.282
TRP 173 (W571)	TYR 2 (Y638)	1	0.087

Hydrogen bonds

Group from Monomer 1	Group from monomer 2	Distance (Å)	Energy (e)
TYR 2 (Y638) OH	THR 171 (T569) OG1	2.43	7.5
SER 4 (S640) OG1	GLN 68 (Q577) NE2	2.18	20.98
GLN 68 (Q577) NE2	SER 4 (S640<9 OG1	2.15	22.43
ARG 159 (R557) O	TPR 173 (W571) NE1	2.05	15.25
THR 171 (T569) OG1	TYR 2 (Y638) OH	2.39	9.03
TPR 173 (W571) NE1	ARG 159 (R557) O	2.07	15.63

(a) Number of contacts involving different carbon atoms (methyl, methylene, methine or aromatic).
(b) Estimated by YASARA software using a knowledge-based potential as described in the software manual. An interaction strength > 5 indicates a strong interaction.
(c) Contacts involving atoms in planar rings.
(d) Interaction strengths range from 0 to 1.
(e) Interaction energy calculated using a simple potential as described in the software manual. Energy ranges from 6.25 (minimun) to 25 kJ/mol (optimum).

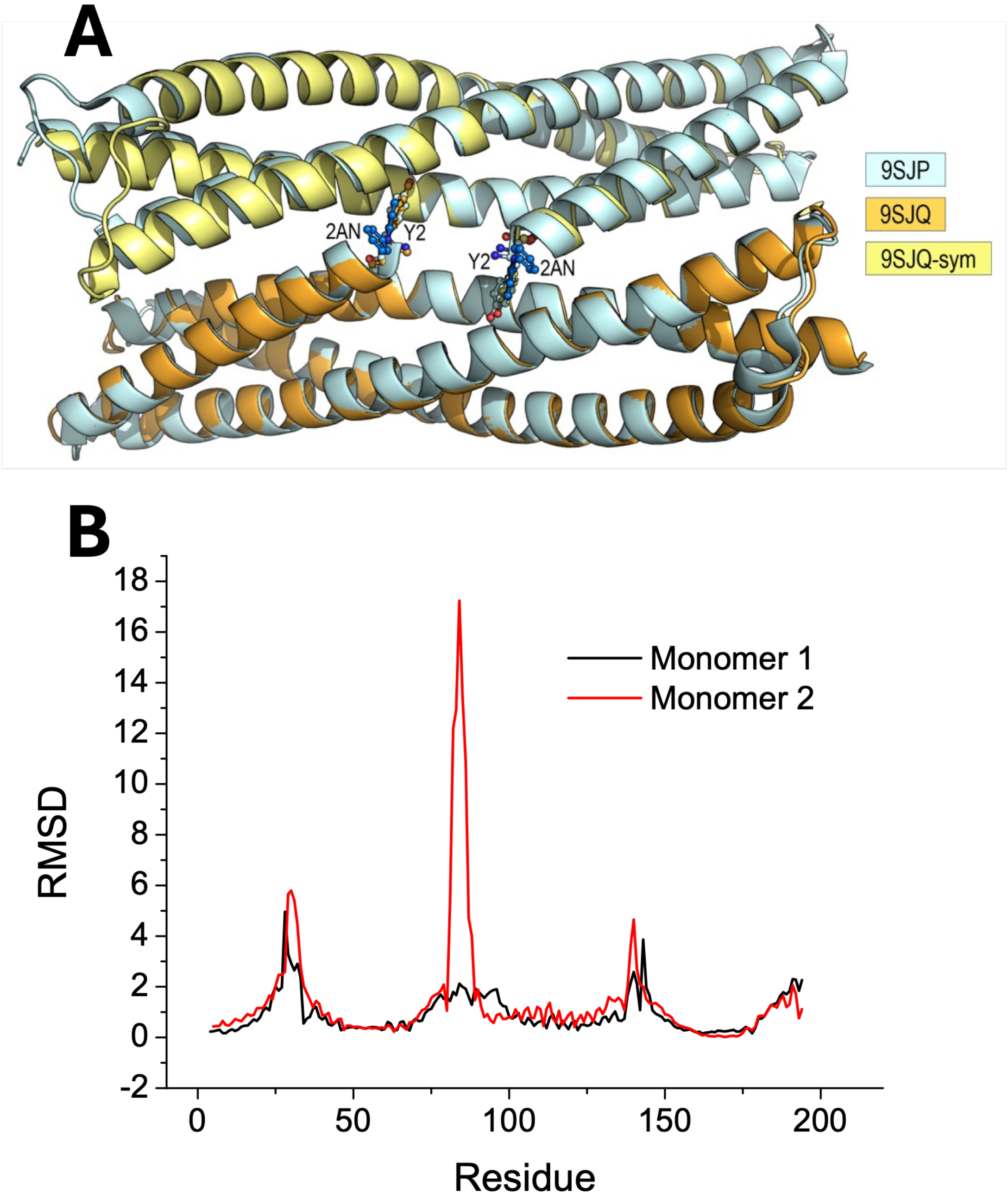

Figure S4. Intermolecular arrangement between Y26L-covNHR chains A and B in the P_1 crystal polymorph and comparison with the $C2_1$ polymorph. A) Ribbon representation of Y26L-covNHR in the P1 crystal polymorph (pale cyan) and its comparison with the C21 polimorph (orange), where the symmetry related molecule 1 has been also represented (yellow). Two ANS molecules and Tyr2 side chains are represented with ball and sticks (ANS in blue and Tyr2 in CPK colors). B) RMSD between the alpha carbons of identical residues of each Y26L-covNHR monomer in the two crystal polymorphs.

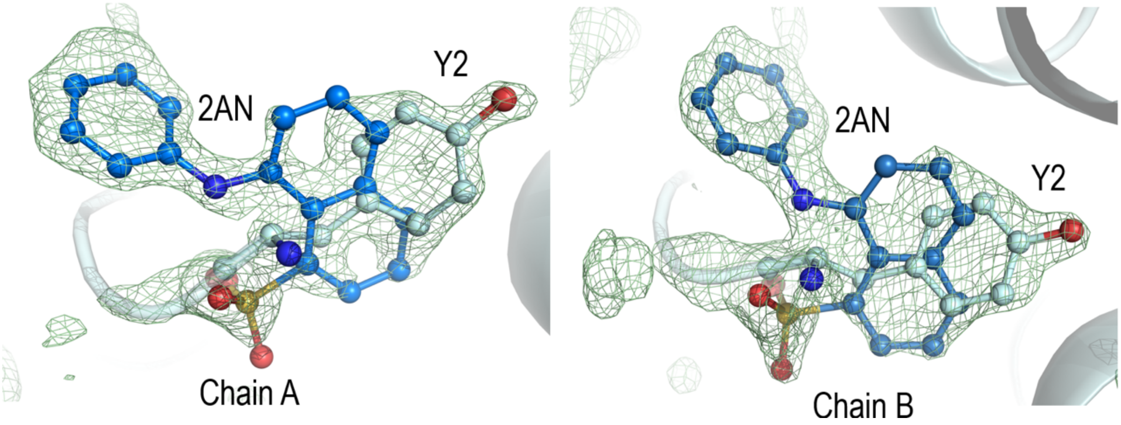

Figure S5. Polder maps for ligand ANS in chain A (left) and B (right). The omit map was contoured to 3σ (green). Y26L-covNHR Tyr2 (Y638) residues (clear cyan) and ANS molecules (blue) are shown in ball and sticks.

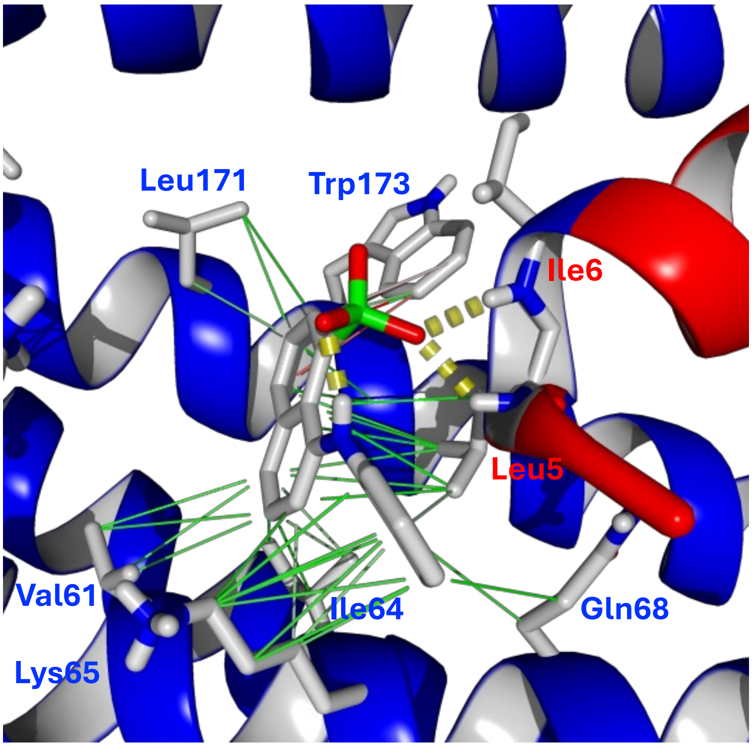

Figure S6. Details of the interactions between ANS and the Y26L-covNHR residues in the P_1 crystal. Hydrogen bonds are represented with yellow dashed lines; hydrophobic contacts are indicated with green lines and π-π stacking interactions with grey lines. ANS and the interacting chimera residues (labelled) are depicted with stick and colored in CPK code. The blue and red ribbons correspond to the backbone of monomer A and monomer B, respectively, in the crystal.

Table S6. Analysis of the interactions between ANS and Y26L-covNHR monomers in the P_1 crystal polymorph

Hydrophobic interactions (cutoff distance: 5 Å)			
Monomer	Residue	Number of contacts [(a)]	Interaction Strength [(b)]
A (HP)	VAL 61 (V570)	3	2.816
A (HP)	ILE 64 (I573)	3	2.449
A (HP)	LYS 65 (K574)	5	6.329
A (HP)	GLN 68 (Q577)	2	1.69
A (HP)	LEU 170 (L568)	3	2.217
A (HP)	TRP 173 (W571)	3	2.31
A (HP)	GLY 174 (G572)	2	1.707
B (CHR)	SER 4 (S640)	1	0.008
B (CHR)	LEU 5 (L641)	7	6.691

π-π interactions (cutoff distance: 5 Å)			
Chain	Residue	Number of contacts [(a)]	Interaction Strength [(b)]
A (HP)	TRP 173 (W571)	3	2.286

Hydrogen bonds			
Group from ANS	Group from monomer 2	Distance (Å)	Energy [(e)]
ANS O3	LEU 5 (L641) N	2.15	11.55
ANS O3	ILE 6 (I642) N	2.04	23.45

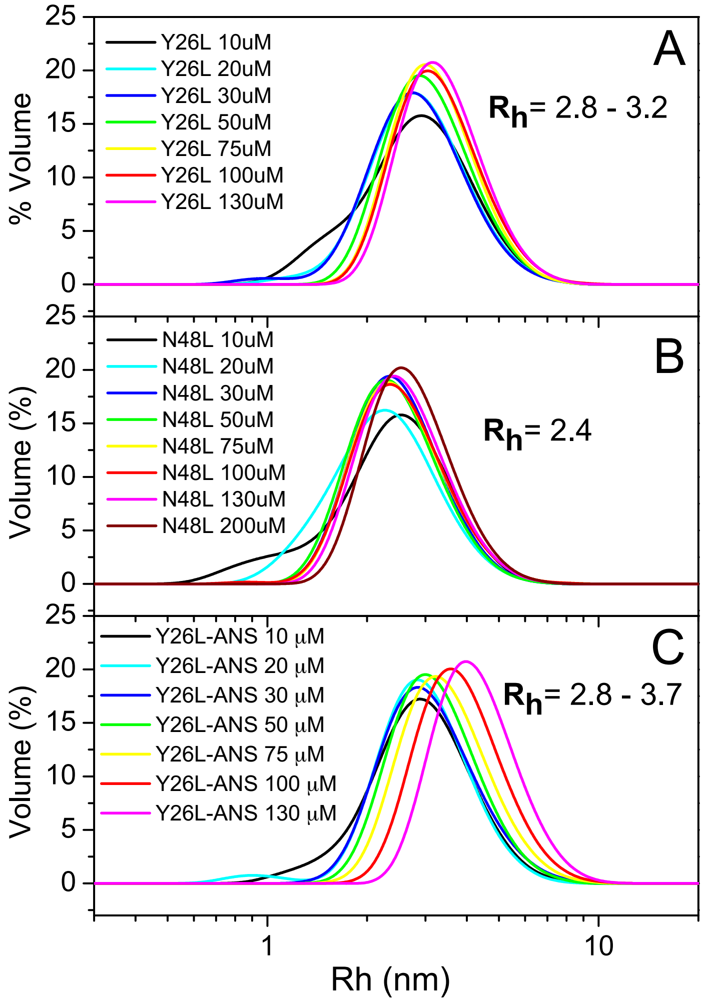

Figure S7. DLS analysis of the molecular size of the chimeras in solution. Hydrodynamic radius distributions of the chimeras measured by dynamic light scattering (DLS) for Y26L-covNHR (A), N48L-covNHR (B) and Y26L-covNHR in presence of 8-fold molar excess of ANS (C) at different protein concentrations as indicated. Experimental conditions are 50 mM sodium phosphate pH 7.4, 25 °C. The range of apparent hydrodynamic radii are shown in nm for each plot.

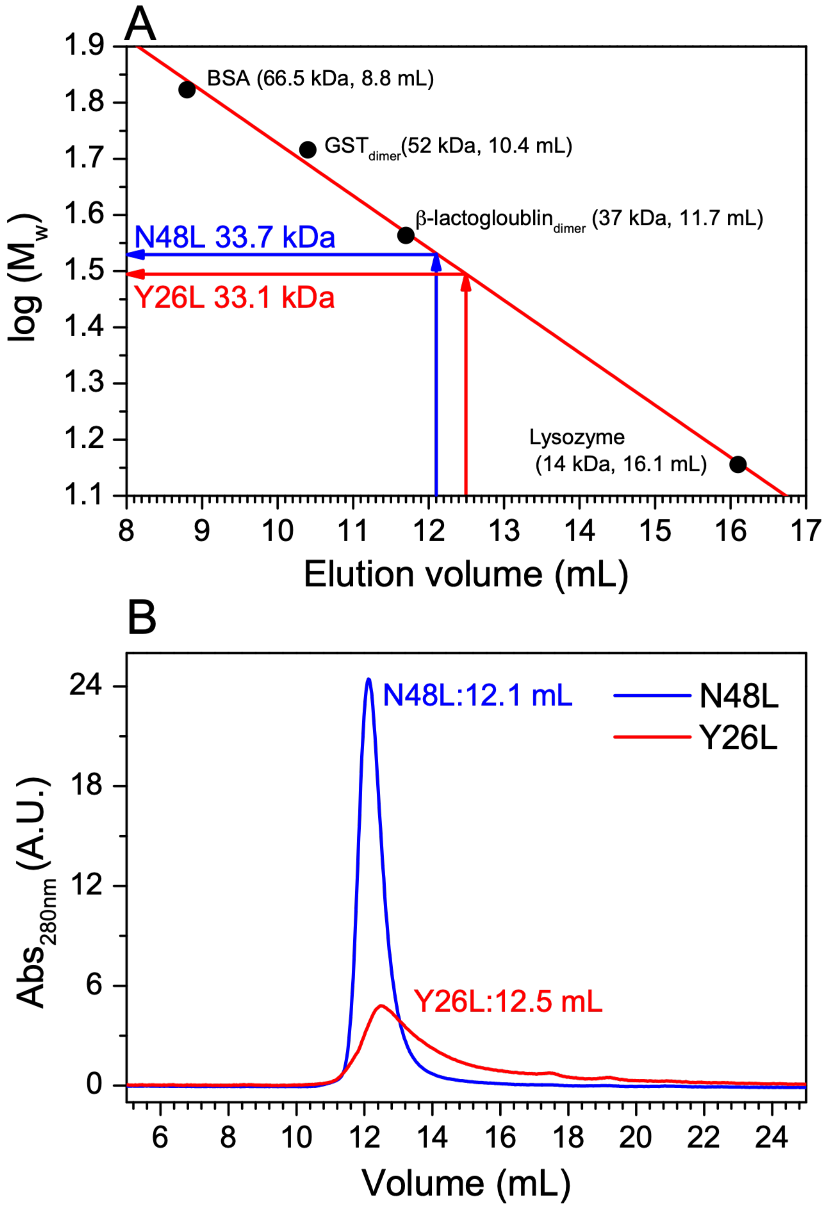

Figure S8. Size-exclusion chromatography (SEC) analysis of the chimeras. Chromatograms were obtained at room temperature using a Superdex 75 10/300 GL column. A) M_w calibration of the SEC column with globular proteins. The elution volumes and the corresponding apparent M_w of the two chimeras are indicated. B) Elution profiles obtained with the two chimeras.

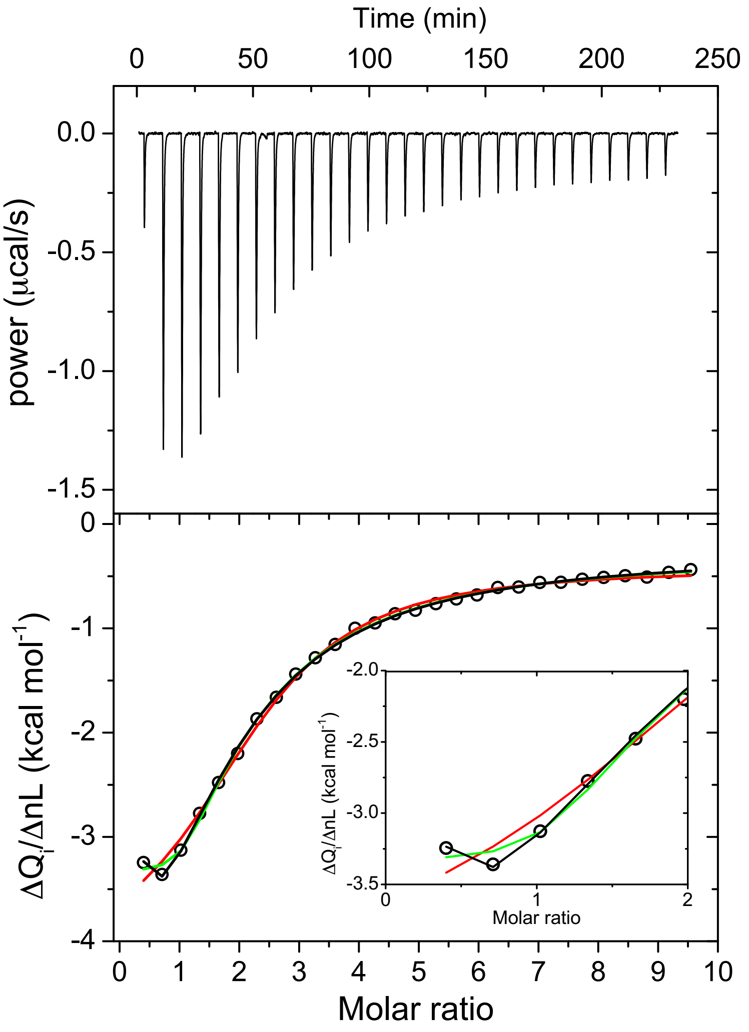

Figure S9. ITC titration of Y26L-covNHR with ANS. Experiment was carried out at 25 °C in 50 mM sodium phosphate buffer. Twentynine injections of 10 µL of 1.26 mM ANS were performed over a 29 µM solution of Y26L-covNHR in the cell. Top: Experimental ITC thermogram corrected from the baseline. Bottom: Binding heats as a function of the molar ANS:chimera ratio. The symbols correspond to the integrated heats normalized per mole of added ANS. The curves represent the best fits using three different binding models: n-independent and identical sites (red); two sequential binding steps (green); binding coupled to dimerization (black). The inset shows the expanded region corresponding to the initial inflexion of the binding curve.

Appendix S2. Mathematical development of the model of ligand binding coupled to dimerization.

We assumed a dimerization equilibrium for a macromolecule, M, with an apparent equilibrium dimerization constant defined as:

$$K_D = \frac{[D]}{[M]^2} = \frac{p_D}{2M_t(1 - p_D)^2}$$

Where p_D is the fraction of macromolecule in the dimeric state. If $p_D = ½$, then:

$$K_D M_t = 1$$

Then, we assumed that the ligand A can bind only to the dimers D, which have 2 identical sites for A with the same microscopic binding constant ***k***. We define a sub-partition function the binding of A to the dimer:

$$Z_A = (1 + k[A])^2$$

Therefore, the binding parameter is:

$$\overline{\nu_A} = \frac{[A]_b}{[D]_T} = \frac{2k[A]}{1 + k[A]}$$

Where the total concentration of dimer, $[D]_T$, can be expressed as:

$$[D]_T = [D](1 + k[A])^2$$

The total concentrations of ligand A and macromolecule M:

$$[A]_T = [A] + [A]_b = [A] + \overline{\nu_A}[D]_T = [A] + [D]\frac{2k[A]}{1 + k[A]}(1 + k[A])^2$$

$$= [A] + 2kK_D[M]^2[A](1 + k[A])$$

$$[M]_T = [M] + 2[D]_T = [M] + 2[D](1 + k[A])^2$$

$$= [M] + 2K_D[M]^2(1 + k[A])^2$$

This set of non-linear equations in [M] and [A] can be solved by Newton-Raphson method, given the total concentrations of M and A and the equilibrium constants.

If we rename: $[A] = x$ and $[M] = z$, we can rewrite the above equations as:

$$F_1(x, z) = x + 2kK_D x z^2(1 + kx) - x_T$$

$$F_2(x,z) = z + 2K_D z^2(1+kx)^2 - z_T$$

Starting from initial estimates of the variables *x* and *z*, the N-R method finds iteratively the increments in the variables *dx* and *dz* that approximate the two functions to zero. In the neighborhood of the optimal values:

$$F_i(x,z) + \frac{\partial F_i}{\partial x} \cdot dx + \frac{\partial F_i}{\partial z} \cdot dz + O(dx^2) + O(dz^2) + \cdots$$

$$\approx F_i + F'_{ix}dx + F'_{iz}dz = 0 \qquad \text{(i = 1, 2)}$$

which leads to the following set of linear equations:

$$F'_{1x}dx + F'_{1z}dz = -F_1$$

$$F'_{2x}dx + F'_{2z}dz = -F_2$$

The derivatives are:

$$F'_{1x} = 1 + 2kK_D z^2(1+kx) + 2k^2K_D xz^2$$

$$F'_{1z} = 4kK_D xz(1+kx)$$

$$F'_{2x} = 4kK_D z^2(1+kx)$$

$$F'_{2z} = 1 + 4K_D z(1+kx)^2$$

We need to solve the system of equations for initial estimates of x and z, to obtain dx and dz, increment the functions and repeat iteratively until convergence. Once converged, using the x = [A] and z = [M] values, we can calculate the system partition function and the fraction of each state.

Taking the free macromolecule monomer, M, as the reference state, the total partition function of the system is:

$$Z = 1 + 2K_D[M](1+k[A])^2 = 1 + 2K_D[M] + 4kK_D[M][A] + 2k^2K_D[M][A]^2$$

The fraction of macromolecule in each state is given by:

$$F_M = \frac{1}{Z} \qquad F_D = \frac{2K_D[M]}{Z}$$

$$F_{DA} = \frac{4kK_D[M][A]}{Z} \qquad F_{DA2} = \frac{2k^2K_D[M][A]^2}{Z}$$

Model formulation to analyze binding experiments by ITC

At constant pressure, the excess enthalpy, relative to the free macromolecule and a certain concentration of free ligand:

$$\Delta H = V_C\{\Delta H_D[D] + (\Delta H_D + \Delta H_A)[DA] + (\Delta H_D + 2\Delta H_A)[DA_2]\}$$

Where V_C is the calorimeter's cell volume in liters, ΔH_D is the enthalpy of dimerization (per mole of dimer) and ΔH_A is the enthalpy of binding of the ligand A to each D site.

After the *ith* ligand injection, the total heat released is:

$$q_i = [M]_T \cdot V_c \cdot \left\{\frac{1}{2}\Delta H_D \cdot F_D + \frac{1}{2}(\Delta H_D + \Delta H_A) \cdot F_{DA} + \left(\frac{1}{2}\Delta H_D + \Delta H_A\right) \cdot F_{DA2}\right\}$$

And the differential heat between two consecutive injections, corrected by the influence of the displaced volume of the cell solution:

$$\Delta q_i = q_i - q_{i-1} = \frac{dV_i}{V_c}\left(\frac{q_i + q_{i-1}}{2}\right)$$

The heat normalized per mole of injected ligand:

$$\frac{\Delta q_i}{\Delta nA} = \frac{\Delta q_i}{dV_i \cdot A_s}$$

Where dV_i is the injected volume in the *ith* injection and A_s is the ligand concentration in the calorimeter's syringe.

Effect of temperature

Since we conducted experiments at different temperatures, we need to consider the heat capacity change ΔC_p of each process. Thus, the enthalpy of dimerization and binding will change with temperature according to:

$$\Delta H_D = \Delta H_D(T_0) + \Delta C_{pD} \cdot (T - T_0)$$

$$\Delta H_A = \Delta H_A(T_0) + \Delta C_{pA} \cdot (T - T_0)$$

And the dimerization and binding constants vary with temperature according to the van't Hoff equation:

$$lnK_D = lnK_D(T_0) - \frac{\Delta H_D(T_0) - \Delta C_{pD} \cdot T_0}{R}\left(\frac{1}{T} - \frac{1}{T_0}\right) + \frac{\Delta C_{pD}}{R} ln\left(\frac{T}{T_0}\right)$$

$$lnk = lnk(T_0) - \frac{\Delta H_A(T_0) - \Delta C_{pA} \cdot T_0}{R}\left(\frac{1}{T} - \frac{1}{T_0}\right) + \frac{\Delta C_{pA}}{R} ln\left(\frac{T}{T_0}\right)$$

Table S7. Thermodynamic parameters of binding of ANS to the Y26L-covNHR chimera measured by ITC using a model of binding coupled to dimerization (see Appendix S2). Values are listed at 25 ºC. Errors correspond to 95% confidence intervals derived from the fit.

K_D [(a)] (M^{-1})	ΔH_D [(b)] (kJ mol^{-1})	k [(c)] (M^{-1})	ΔH_A [(d)] (kJ·mol^{-1})	ΔC_{pA} [(d)] (kJ·K^{-1}·mol^{-1})
$(8 \pm 2) \times 10^3$	60 ± 10	$(7.5 \pm 0.9) \times 10^4$	-61 ± 3	0.48 ± 0.18

(a)Equilibrium dimerization constant

(b)Enthalpy of dimerization (per mol of dimer)

(c)ANS binding constant

(d)ANS binding enthalpy

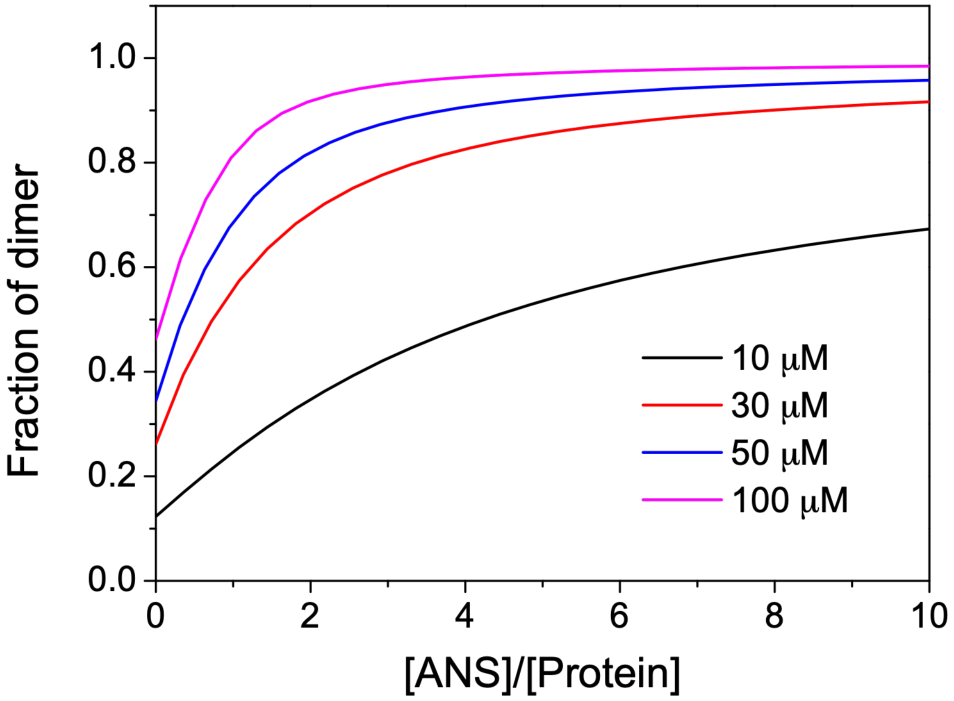

Figure S10. Fraction of Y26L-covNHR in the dimeric state as a function of the ANS/protein molar ratio. The curves have been calculated at 25 °C at different protein concentrations as indicated in different colors, using the mathematical model of Appendix S2 and the thermodynamic parameters listed in Table S7.

Supplementary references

1. Luo P, Baldwin RL (1997) Mechanism of Helix Induction by Trifluoroethanol: A Framework for Extrapolating the Helix-Forming Properties of Peptides from Trifluoroethanol/Water Mixtures Back to Water. Biochemistry 36:8413–8421.

2. Jurado S, Cano-Muñoz M, Morel B, Standoli S, Santarossa E, Moog C, Schmidt S, Laumond G, Cámara-Artigas A, Conejero-Lara F (2019) Structural and Thermodynamic Analysis of HIV-1 Fusion Inhibition Using Small gp41 Mimetic Proteins. J. Mol. Biol. 431:3091–3106.

3. Krieger E, Vriend G (2014) YASARA View—molecular graphics for all devices—from smartphones to workstations. Bioinformatics 30:2981–2982.
